# Semigroup models for biochemical reaction networks

**DOI:** 10.1007/s00285-023-01898-5

**Published:** 2023-04-19

**Authors:** Dimitri Loutchko

**Affiliations:** Institute of Industrial Science, The University of Tokyo, 4-6-1, Komaba, Meguro-ku, Tokyo, 153-8505 Japan

**Keywords:** Biochemical reaction networks, Autocatalytic sets, Algebraic models, Finite semigroups, 20M35, 92E20

## Abstract

The catalytic reaction system (CRS) formalism by Hordijk and Steel is a versatile method to model autocatalytic biochemical reaction networks. It is particularly suited, and has been widely used, to study self-sustainment and self-generation properties. Its distinguishing feature is the explicit assignment of a catalytic function to chemicals that are part of the system. In this work, it is shown that the subsequent and simultaneous catalytic functions give rise to an algebraic structure of a semigroup with the additional compatible operation of idempotent addition and a partial order. The aim of this article is to demonstrate that such semigroup models are a natural setup to describe and analyze self-sustaining CRS. The basic algebraic properties of the models are established and the notion of the function of any set of chemicals on the whole CRS is made precise. This leads to a natural discrete dynamical system on the power set of chemicals, which is obtained by iteratively considering the self-action on a set of chemicals by its own function. The fixed points of this dynamical system are proven to correspond to self-sustaining sets of chemicals, which are functionally closed. Finally, as the main application, a theorem on the maximal self-sustaining set and a structure theorem on the set of functionally closed self-sustaining sets of chemicals are proven.

## Introduction

Understanding and adequately modeling the organizational structure of biochemical reaction networks is an exciting area of modern research. There is one crucial property which distinguishes, on a holistic level, reaction networks encountered in real biological systems from arbitrary ones, namely their ability to *self-generate*, *self-sustain* or *self-replicate*. Initiated by von Neumann (1966) and his theory of self-replicating automata, several holistic approaches have been proposed with the primary aim of capturing general and universal properties of biochemical reaction networks, with a particular focus on the functional organization and self-sustainment, self-generation, and self-replication properties of the reaction networks of living and evolving systems. Important contributions include (*M*, *R*)-systems by Rosen (1958), hypercycles introduced by Eigen (1971), autopoetic systems studied by Varela et al. (1974), chemotons by Gánti (1975), autocatalytic sets by Kauffman (1986) and catalytic reaction systems developed by Hordijk and Steel (2004). The common property of such models is that they focus on the catalysis of network reactions by chemicals which are themselves produced by reactions within the network. They usually do not require kinetic details, but only the knowledge of the occurring reactions, together with data on catalysis. An in-depth discussion and comparison of such approaches was given by Hordijk and Steel (2018).

The construction of the semigroup models presented in this article is based on the formalism of catalytic reaction systems (CRS) introduced by Hordijk and Steel (2004, 2018); Hordijk et al. (2011). The CRS formalism is a generalization of autocatalytic sets by Kauffman (1971, 1986, 1993) and is broad enough to encompass several of the aforementioned approaches. The seminal work by Kauffman (1971, 1986, 1993) takes inspiration from polymer-based approaches to the early stages of evolution such as the RNA world hypothesis or protein-based metabolism-first models. The RNA world hypothesis states that RNA molecules might have been responsible for the catalysis of both replication and biosynthesis reactions in the early stages of evolution, as formulated by Gilbert (1986), Joyce (1989), and many others. Protein-based metabolism-first approaches are similar in spirit but focus on polypeptides instead of RNA and are discussed, for example, by Vasas et al. (2012). Kauffman studies sets of polymers, each of which has a certain probability to catalyze any ligation or cleavage reaction, and finds that there is threshold for this probability above which self-generating sets of polymers almost surely appear. Kauffman’s work is valuable for theoretical biology because it provides a mathematical foundation for the investigation of polymer-based approaches, albeit the probability of catalysis being a virtually inaccessible parameter. A more general approach to the origin of life has been proposed by Oparin (1957) and later popularized by Dyson (1999). Oparin’s theory is, in spirit similar to polymer-based approaches in the sense that he proposes self-generating and self-sustaining chemical reaction networks as the precursors to modern biochemical assemblies. However, he suggests that such networks need not be composed of polymers but can contain arbitrary chemicals—organic and inorganic, homogeneous and heterogeneous—which can act as catalysts for the reactions of the network. The CRS formalism generalizes Kauffman’s framework to encompass Oparin’s general setting in the sense that it admits arbitrary chemicals instead of polymers. One of the earlier results consists in finding and analyzing the threshold probabilities for the occurrence of self-sustaining sets of chemical reaction systems by Hordijk and Steel (2004, 2017), akin to Kauffman’s results on polymer networks; in addition, non-uniform catalysis probability distributions have been analyzed by Hordijk et al. (2014) and the effect of molecular diversity has been investigated by Hordijk et al. (2019). More importantly, the more variable CRS approach allows to apply the formalism to actual biochemical reaction networks. This provides an understanding of their modular and hierarchical compositions and of the structure of autocatalytic motives which even leads to valuable information on the evolution of the system under investigation. Such work was carried out by Sousa et al. (2015), Xavier et al. (2020) and Xavier and Kauffman (2022).

The main purpose of this article is to flesh out the algebraic structure which is inherent in the CRS formalism and to investigate the properties of the resulting semigroup models. In particular, the construction allows to make precise the notion of the catalytic function of chemicals and of sets of chemicals on the whole CRS. Such constructions have implicitly appeared in various algorithms by Hordijk and Steel (2004); Hordijk et al. (2015) for the detection of self-sustaining sets in the literature. However, their inherent algebraic nature has not been formalized thus far. In order to illustrate that the semigroup models provide a well-suited mathematical language for CRS, it is shown how self-sustaining subsets of chemicals can be characterized in a concise manner and how the largest self-sustaining subset of chemicals can be determined for any CRS. Moreover, within the semigroup formalism it is natural to consider self-sustaining sets of chemicals which cannot produce chemicals not already contained in the respective set. Such sets have not been considered in the CRS literature thus far and the term *functionally closed sets* of chemicals is coined here for them. This is conceptually related to the closure property in chemical organization theory, see the review by Dittrich and Di Fenizio (2007). The set of all functionally closed sets of the CRS is characterized and its potential applications to the analysis of CRS which model real biological systems are discussed. In a nutshell, the semigroup models provide natural tools to concisely state and solve problems in CRS theory and to find new meaningful constructions within the theory.

Besides their applications to CRS theory, semigroup models open up the field of biochemical reaction networks to be tackled with algebraic methods. For example, the equivalence of finite semigroups and finite automata suggests to investigate the computational capabilities of chemical reaction networks from this abstract point of view. Moreover, it will be interesting and rewarding to construct a purely algebraic description of semigroup models without the need to consider their representation as maps on a finite set. One step in this direction, which is proven in this work, is the fact that the semigroup models of CRS with a nontrivial self-sustaining sets of chemicals cannot be nilpotent. This excludes a large class of potential semigroup models to be considered in inverse problems in the future.

***Mathematical outline***

This outline summarizes the important constructions and the main results of this article. To illustrate the concepts and to make them more accessible to readers with a background in biology rather than mathematics, a toy example is provided in “Appendix A”, where all constructions are carried out explicitly and the intuition behind them is explained.

**The CRS formalism** In Sect. 2, the CRS formalism is introduced following Hordijk and Steel (2004, 2017). A CRS uses the datum of a chemical reaction network, i.e. a finite set of chemicals *X* together with a finite set of net chemical reactions *R*. In addition, a set of catalysis data $C \subset X \times R$ is specified by stipulating that for each $(x,r) \in C$, the reaction *r* is catalyzed by the chemical *x*. Finally, a subset $F \subset X$, called food set, of constantly supplied chemicals is given. The kinetic rate constants of the chemical reaction network are not part of the datum of a CRS. Within the CRS formalism, the notion of self-sustainment has been formalized under the name of pseudo-RAF by Steel (2015) and Hordijk and Steel (2017).

**The semigroup model of a CRS** In Sect. 3.1, the catalytic function of chemicals is given the structure of a semigroup, which is additionally equipped with a partial order and an idempotent addition. In spirit, this construction is motivated by the work of Rhodes and Nehaniv (2010). There are, however, substantial technical differences, most notably the lack of addition and partial order, as discussed in Remark 3.4.

To begin with, to each reaction $r \in R$, a function $\phi _r$ is assigned as the set-map $\phi _r: {\mathfrak {X}} \rightarrow {\mathfrak {X}}$ on the power set ${\mathfrak {X}}:={\mathcal {P}}(X_F)$ of non-food chemicals $X_F = X {\setminus } F$. The function $\phi _r$ gives the set of non-food products of the reaction *r* if and only if the set of non-food reactants is contained in its argument. Such functions have an idempotent addition via $(\phi _r + \phi _{r'})(Y) = \phi _r(Y) \cup \phi _{r'}(Y)$ for any $Y \subset X_F$ and any $r,r' \in R$. The function $\phi _x$ of a chemical $x \in X$ is defined as the sum of the reactions catalyzed by it:

$$\begin{aligned} \phi _x = \sum _{(x,r) \in C} \phi _r. \end{aligned}$$

The semigroup model

$$\begin{aligned} {\mathcal {S}} = \langle \phi _x \rangle _{x\in X} \end{aligned}$$

of a CRS is generated by the functions $\{\phi _x\}_{x\in X}$ through addition $+$ and through the composition of functions $\circ$. Thus, ${\mathcal {S}}$ is a semigroup with respect to both $+$ and $\circ$ and hence it is called a *semigroup model* of the CRS. For any subset $Y \subset X_F$, the semigroup model ${\mathcal {S}}(Y)$ is defined as ${\mathcal {S}}(Y) = \langle \phi _x \rangle _{x\in Y \cup F}$. Moreover, ${\mathcal {S}}$ is endowed with a partial order given by $\phi \le \psi$ iff $\phi (Y) \subset \psi (Y)$ for all $Y \subset X_F$. The unique maximal element of ${\mathcal {S}}(Y)$ is denoted by $\Phi _Y$—it represents all catalytic functionality that can be exhibited by the set *Y* on the whole CRS. In Sect. 3.2, elementary properties of the semigroup models are established. In a nutshell, these properties ensure that the two operations and the partial order on ${\mathcal {S}}$ are compatible.

**Characterization of self-sustaining CRS** In Sect. 4, the semigroup models are used to derive theorems characterizing self-sustaining CRS and subCRS and their corresponding sets of chemicals. Theorem 4.1 concisely states that a CRS is self-sustaining if and only if the set of chemicals $X_F$ is able to fully reproduce itself. In the semigroup formalism this reads as

$$\begin{aligned} \Phi _{X_F}(X_F) = X_F. \end{aligned}$$

It is then shown that each self-sustaining set of chemicals $X'_F \subset X_F$ satisfies $X'_F \subset \Phi _{X'_F}(X'_F)$ (Corollary 4.2) and that the equality $X'_F = \Phi _{X'_F}(X'_F)$ is a sufficient condition to be a self-sustaining set of chemicals (Proposition 4.3). Finally, a discrete dynamics on the state space ${\mathfrak {X}}$ is defined by the self-action of the chemicals on themselves, i.e. by $Y \mapsto \Phi _Y(Y)$ for $Y \in {\mathfrak {X}}$. It is shown that the dynamics with the initial condition $X_F$ leads to a fixed point, denoted by $X_F^{*s}$ and, using a combination of the tools developed in this article, it is proven that the maximal self-sustaining set of chemicals of any CRS is given by this fixed point (Theorem 4.14).

**Functionally closed self-sustaining sets of chemicals** In Sect. 4.4, new concepts are introduced into CRS theory, which are motivated by the tools from the preceding sections. The containment of a set of chemicals $X_F'$ in the set of chemicals which it generates, i.e. $X_F' \subset \Phi _{X'_F}(X'_F)$, suggests that such sets should not be observable in real biological systems. Instead, they will continue to produce chemicals according to the dynamics ${X_F' =: Y_0 \mapsto \Phi _{Y_0}(Y_0) =: Y_1 \mapsto \Phi _{Y_1}(Y_1) \mapsto \cdots }$, which is shown to stabilize at the self-sustaining set of chemicals $X_F'^{*s}$ (Proposition 4.17). The set $X_F'^{*s}$ is called the *functional closure* of $X_F'$ and it is conjectured that within a CRS such sets comprise biologically relevant functional modules of a CRS. Sets $X_F'$ which satisfy $X_F' = \Phi _{X'_F}(X'_F)$ are called *functionally closed* and the ones which satisfy $X_F' \subset \Phi _{X'_F}(X'_F)$ are termed *pre-functionally closed*. To determine the set of all (pre-)functionally closed sets of chemicals, a reduced dynamics on ${\mathfrak {X}}$ is introduced via $Y \mapsto Y \cap \Phi _Y(Y)$. This dynamics has a fixed point $Y_0^{*rs}$ for any initial condition $Y_0 \in {\mathfrak {X}}$. The set $\mathfrak {pF}$ of pre-functionally closed sets of chemicals is given by

$$\begin{aligned} \mathfrak {pF}:= \bigcup _{i=0}^{\mid X_F^{*s} \mid } \mathfrak {pF}^{i} \subset {\mathfrak {X}}, \end{aligned}$$

where the $\mathfrak {pF}^i$ are defined by the recursion

$$\begin{aligned} \mathfrak {pF}^0&:= \{ X_F^{*s} \} \\ \mathfrak {pF}^{i+1}&:= \bigcup _{Y \in \mathfrak {pF}^{i}} {\mathfrak {p}}(Y) \end{aligned}$$

for $i \ge 0$ and where the map ${\mathfrak {p}}: {\mathfrak {X}} \rightarrow {\mathcal {P}}({\mathfrak {X}})$ is given by

$$\begin{aligned} {\mathfrak {p}}(Y):= \left\{ (Y{\setminus }\{y\})^{*rs} \right\} _{y \in Y} \subset {\mathfrak {X}} \end{aligned}$$

for any $Y \in {\mathfrak {X}}$. This is proven in Theorem 4.26 and provides an explicit description, which can be algorithmically implemented, of $\mathfrak {pF}$. The desired set ${\mathfrak {F}}$ of functionally closed sets of chemicals is the subset of $\mathfrak {pF}$ given by ${\mathfrak {F}} = \{Y \in \mathfrak {pF} \text { such that } Y = \Phi _Y(Y)\}$. Lemma 4.20 gives another characterization of ${\mathfrak {F}}$ as the set of the functional closures of all elements of $\mathfrak {pF}$, i.e.,

$$\begin{aligned} {\mathfrak {F}} = \{ Y^{*s} \text { for } Y \in \mathfrak {pF} \}. \end{aligned}$$

Finally, it is important to establish the connection between the concept of self-sustaining sets of chemicals and (pre-)functionally closed sets of chemicals. According to Corollary 4.2 pre-functional closure is a necessary condition for self-sustainment and by Proposition 4.3 functional closure is sufficient for self-sustainment. Therefore, the following inclusions hold trueThis is the content of Theorem 4.21. In the final part of Sect. 4.4, biochemical implications the new notions are discussed and possible future mathematical constructions are sketched.

This finishes the exposition of the main contents of this article. In a companion article by Loutchko (2023), the self-generating properties of CRS are investigated in an analogous fashion. Although the logical structure of the line of reasoning is analogous to the one presented here, the proofs themselves require more technical setup, which heavily relies on a representation of the semigroup elements as decorated rooted trees. In this regard, Sect. 4 of this article can be seen as a blueprint to the work in Loutchko (2023). The current article is aimed as an introduction to semigroup models of CRS, whereas the companion article is focused on their structure and provides more technical tools.

## The CRS formalism

### Basic notions

The catalytic reaction system (CRS) formalism is introduced following Hordijk and Steel (2004). A chemical reaction network is a finite set of chemicals *X* together with a finite set of net chemical reactions *R*. Thereby, a reaction $r \in R$ is given by a pair of sets $r=(\text {dom}(r),\text {ran}(r))$ which satisfy $\text {dom}(r),\text {ran}(r) \subset X$ and $\text {dom}(r) \cap \text {ran}(r) = \emptyset$ and are called the *domain* and the *range* of the reaction.^1^ The latter condition ensures that one is dealing with a *net* chemical reaction. Catalysis and autocatalysis is treated in Definition 2.1 below by giving additional data on the catalyzed reactions. The support $\text {supp}(r)$ of a reaction is defined as

$$\begin{aligned} \text {supp}(r) = \text {dom}(r) \cup \text {ran}(r). \end{aligned}$$

In addition, for a set of reactions $R' \subset R$, the domain, range and support are defined as

$$\begin{aligned} \text {dom}(R')&= \bigcup _{r \in R'} \text {dom}(r), \\ \text {ran}(R')&= \bigcup _{r \in R'} \text {ran}(r), \\ \text {supp}(R')&= \bigcup _{r \in R'} \text {supp}(r) = \text {dom}(R') \cup \text {ran}(R'). \end{aligned}$$

Classically, a set of rate constants together with the stoichiometry of each $r \in R$ would lead to a system of coupled ordinary differential equations for the time evolution for the concentration of each chemical $x \in X$ given by a kinetic model such as mass action kinetics. However, the CRS formalism does not utilize this detailed kinetic information but instead emphasizes the catalytic function of the chemicals in *X*. Moreover, a food set $F \subset X$ is specified. This set is to be thought of as the set of externally supplied chemicals, which are always readily available to the system. The abbreviation

$$\begin{aligned} X_F:= X {\setminus } F \end{aligned}$$

for the set of all non-food chemicals will be used. Often, a subset of $X_F$ will be denoted by $X'_F$, which implicitly carries the information of the corresponding subset $X' = X'_F \cup F$ of *X*.

#### Definition 2.1

A *catalytic reaction system* (CRS) is a tuple (*X*, *R*, *C*, *F*) where *X* is a finite set of chemicals, *R* is a finite set of reactions, $C \subset X \times R$ is the catalysis data, and $F \subset X$ is the constantly present food set. For a pair $(x,r) \in C$, the reaction *r* is said to be catalyzed by *x*. The food set is required to satisfy the following closure property:

(C) All reactions $r \in R$ with a catalyst in *F* must involve at least one reactant outside of *F*, i.e. they must satisfy $\text {dom}(r) \cap X_F \ne \emptyset$.

If $X=F$, the CRS is said to be *trivial*.

It is convenient to introduce the projection

2.1 $$\begin{aligned} \pi _R&: C \rightarrow R, \\ \nonumber&(x,r) \mapsto r. \end{aligned}$$

The image $\pi _R(C)$ gives all catalyzed reactions of the CRS.

#### Remark 2.2

In the CRS literature it is common to consider the triple (*X*, *R*, *C*) as the definition of a CRS and to give the food set $F \subset X$ as an additional datum, cf. Hordijk and Steel (2004); Hordijk et al. (2011). The tuple (*X*, *R*, *C*, *F*) from Definition 2.1 would then be called a *CRS with food set*. Because this article is concerned with tuples (*X*, *R*, *C*, *F*) exclusively, they are simply called CRS for the sake of readability. The closure condition (C) is not commonly used in the CRS literature. However, it poses no serious restrictions but merely serves to exclude trivial cases of self-sustainment, wherein chemicals in $X_F$ are produced purely from the food set by reactions with catalysts in the food set.

**Fig. 1:**
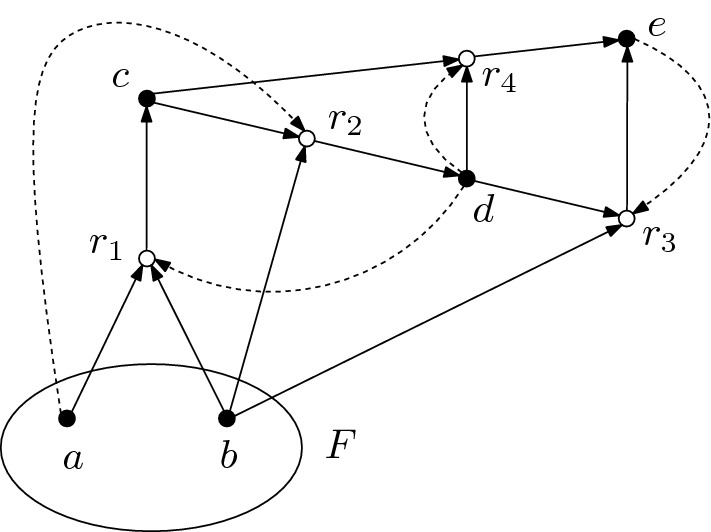
Example of a graphical representation of a CRS. The CRS consists of five chemicals $X=\{a,b,c,d,e\}$ and four reactions $a+b\rightarrow c$, $c+b \rightarrow d$, $b+d \rightarrow e$ and $c+d \rightarrow e$, which are catalyzed by *d*, *a*, *e* and *d*, respectively. The food set is given by $F = \{a,b\}$

Following Bonchev and Mekenyan (2012), a CRS can be represented as a directed bipartite graph with a partition of the edges into edges corresponding to reactions and edges corresponding to catalysis. As an example, consider the graph in Fig. 1. The vertices represented by solid disks correspond to the chemicals *X* and the vertices represented by circles correspond to reactions *R*. Solid directed edges from chemicals to a reaction indicate that the respective chemicals are the reactant of the reaction, whereas solid directed edges from a reaction to chemicals are present for the products of the respective reaction. Dashed edges from a chemical *x* to a reaction *r* represent elements (*x*, *r*) in the catalysis data *C*. The food set is indicated by a circle, which encloses the chemicals belonging it. The stoichiometry of the reactions is not shown in the graph.

#### Definition 2.3

A tuple $(X',R',C',F)$ is said to be a *subCRS* of the full CRS (*X*, *R*, *C*, *F*) if the following properties are satisfied

1. $$X' \subset X$$,
2. The food set $F \subset X'$ is identical to the one of the full CRS.
3. $$R' \subset R\mid _{X'}$$,
4. $$C' = C \cap (X' \times R') \subset C\mid _{X'}$$,

where the restrictions $R\mid _{X'}$ and $C\mid _{X'}$ are given by

$$\begin{aligned} R\mid _{X'}&= \{r \in R \text { with supp}(r) \subset X'\}, \\ C\mid _{X'}&= C \cap (X' \times R\mid _{X'}). \end{aligned}$$

The sets $R\mid _{X'}$ and $C\mid _{X'}$ represent the maximal set of reactions supported on $X'$ and the maximal catalysis data with reactions supported on $X'$ and catalysts in $X'$, respectively.

Note that a subCRS is determined by solely the two subsets $X' \subset X$ and $R' \subset R\mid _{X'}$. The set $C'$ is the maximal catalysis data for reactions in $R'$ that are catalyzed by some chemical in $X'$. A subCSR $(X',R',C',F)$ always satisfies condition (C): By Definition 2.3, a reaction $r \in R'$ satisfies $\text {dom}(r) \subset X'$ and therefore $\text {dom}(r) \cap X'_F = \text {dom}(r) \cap X_F$ holds. If it has a catalyst in *F*, then $\text {dom}(r) \cap X_F \ne \emptyset$ holds by condition (C) for the full CRS (*X*, *R*, *C*, *F*), which implies $\text {dom}(r) \cap X'_F \ne \emptyset$. Therefore, a subCRS is always a CRS.

The subCRS of a CRS are ordered by inclusion, i.e. two subCRS are related by $(X',R',C',F) \le (X'',R'',C'',F)$ iff $X' \subset X''$ and $R' \subset R''$ are satisfied. For a set of non-food chemicals $X'_F \subset X_F$, the maximal subCRS with the set of chemicals $X'$ is given by $(X',R\mid _{X'},C\mid _{X'},F)$. It is called the *subCRS generated by* $X'_F$ and denoted by

2.2 $$\begin{aligned} CRS(X'_F):= (X',R\mid _{X'},C\mid _{X'},F). \end{aligned}$$

### Catalytic and self-sustainment properties

Self-sustaining CRS are characterized by the property that all non-food chemicals are products of catalyzed reactions which use only chemicals produced by the reactions themselves and the food set. Self-sustaining CRS have been discussed in Hordijk and Steel (2017) and Steel (2015), where they are called pseudo-RAF. In this section, self-sustaining CRS and, based on them, self-sustaining sets of chemicals are defined. Finally, the precise connection to the definition of pseudo-RAF as given by Hordijk and Steel (2017) is discussed.

#### Definition 2.4

A CRS (*X*, *R*, *C*, *F*) is called *self-sustaining* if the following condition is satisfied:

(S) There exists a set of reactions $R' \subset \pi _R(C)$ such that $X_F \subset \text {ran}(R')$.

Note that it is not necessary that all reactions in *R* are catalyzed but only that the CRS has enough catalyzed reactions to satisfy condition (S). A subCRS is said to be self-sustaining if it satisfies the Definition 2.4. For a given set of chemicals $X' \subset X$, the level of catalysis can differ between subCRS which have $X'$ as the set of chemicals. In this regard, the following notion is useful:

#### Definition 2.5

A subCRS $(X',R',C',F)$ is *closed* if its set of reactions satisfies

$$\begin{aligned} \pi _{R'}(C') = \pi _{R\mid _{X'}}(C\mid _{X'}). \end{aligned}$$

In other words, if and only if all catalyzed reactions of the full CRS (*X*, *R*, *C*, *F*) which have a catalyst and support in $X'$ are included in $R'$, then the respective subCRS is closed. Note that the full CRS (*X*, *R*, *C*, *F*) is closed by definition.

Moreover, it has a maximal, possibly trivial, self-sustaining subCRS given by the union of all its self-sustaining subCRS (the union of two given subCRS is the smallest subCRS for which both are subCRS). This maximal self-sustaining subCRS is necessarily closed.

A set of chemicals $X'_F \subset X_F$ generates $CRS(X'_F)$ via formula (2.2) and thus the notion self-sustainment, as introduced in Definition 2.4, is inherited by it:

#### Definition 2.6

A set of chemicals $X'_F \subset X_F$ is said to be a *self-sustaining set of chemicals* if the maximal subCRS generated by it, $CRS(X'_F)$, is self-sustaining. The empty set $\emptyset \subset X_F$ is said to be the trivial self-sustaining set of chemicals.

If $X'_F$ is a self-sustaining set of chemicals, then $CRS(X'_F)$ is automatically closed due to the maximality of $R\mid _{X'}$ and $C\mid _{X'}$. Therefore, the notion of self-sustainment on the level of chemicals does not encompass non-closed CRS.

#### Example 2.7

**Fig. 2:**
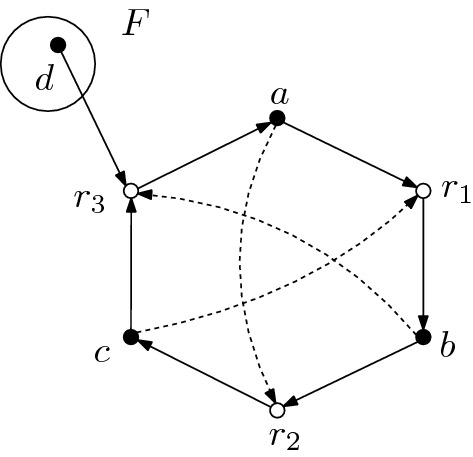
An example of a self-sustaining CRS

The CRS in Fig. 2  is self-sustaining in the sense that all non-food chemicals $X_F = \{a,b,c\}$ are produced by the set of reactions $R =\{r_1,r_2,r_3\}$. The set of reactions satisfies $X_F \subset \text {ran}(R) = \{a,b,c\}$ and $\text {dom}(R) = \{a,b,c,d\} \subset \text {ran}(R) \cup F = \{a,b,c,d\}$. Notice that, in this example, none of the chemicals can be generated from the food set alone.

#### Remark 2.8

(Relation to the notion of pseudo-RAF commonly used in the literature) The definition of a pseudo-RAF given by Hordijk and Steel (2017) (see also Steel et al. (2013); Steel (2015); Hordijk et al. (2015)) is based on a set $R' \subset \pi _R(C)$ of catalyzed reactions. More precisely, a subset $R' \subset \pi _R(C)$ is called pseudo-RAF if $\text {dom}(R') \subset \text {ran}(R') \cup F$ holds. Therefore, given a subCRS $(X',R',C',F)$, which is self-sustaining according to Definition 2.4, the set $\pi _{R'}(C')$ is a pseudo-RAF set of reactions. *Vice versa*, given a pseudo-RAF set of reactions $R' \subset R$, the subCRS $(X',R',C',F)$ with $X':= \text {supp}(R')$ and $C':= C \cap ( X' \times R')$ is self-sustaining according to Definition 2.4. Therefore, both definitions are equivalent modulo the inclusion of uncatalyzed reactions in the sets of reactions in the definition used in this article.^2^

The self-sustaining sets of chemicals introduced in Definition 2.6 correspond to closed pseudo-RAF sets of reactions via $X'_F \mapsto R\mid _{X'}$ and, *vice versa*, a closed pseudo-RAF set of reactions $R'$ yields the corresponding self-sustaining set of chemicals as $X'_F:= \text {supp}(R') \cap X_F$.

## The semigroup model of a CRS

The catalytic function of the chemicals gives rise to an algebraic structure generated by the simultaneous and subsequent catalytic functionality. In this section, this structure is formalized mathematically and leads to the notion of a semigroup model of a CRS. Throughout this section, let the CRS (*X*, *R*, *C*, *F*) be fixed.

### Construction

The state of the CRS is defined by the presence or absence of each of the non-food chemicals, i.e. by a subset $Y \subset X_F$ (the chemicals in the food set are always present). Thus the state space ${\mathfrak {X}}$ of the CRS is defined to be the power set of $X_F$

$$\begin{aligned} {\mathfrak {X}} := {\mathcal {P}}(X_F). \end{aligned}$$

The function of a given chemical $x \in X$ is defined via the reactions it catalyzes, i.e. by the way it acts on the state space ${\mathfrak {X}}$. The following definition is inspired by the work of Rhodes and Nehaniv (2010).

#### Definition 3.1

Let (*X*, *R*, *C*, *F*) be a CRS with food set. The *function* $\phi _r$ of a reaction $r \in R$ is the map

$$\begin{aligned} \phi _r: {\mathfrak {X}} \rightarrow {\mathfrak {X}} \end{aligned}$$

given by

3.1 $$\begin{aligned} \phi _r(Y) ={\left\{ \begin{array}{ll} \text {ran}(r) \cap X_F &{} \text {if } \text {dom}(r) \subset Y \cup F \\ \emptyset &{} \text {else} \end{array}\right. } \end{aligned}$$

for all $$Y \subset X_F$$.^3^

For any two maps $\phi , \psi : {\mathfrak {X}} \rightarrow {\mathfrak {X}}$, the sum $(\phi + \psi ): {\mathfrak {X}} \rightarrow {\mathfrak {X}}$ and product $(\phi \circ \psi ): {\mathfrak {X}} \rightarrow {\mathfrak {X}}$ are given by

3.2 $$\begin{aligned} (\phi + \psi )(Y)&:= \phi (Y) \cup \psi (Y) \end{aligned}$$

3.3 $$\begin{aligned} (\phi \circ \psi )(Y)&:= \phi (\psi (Y)) \end{aligned}$$

for all $Y \subset X_F$. The *function* $\phi _x: {\mathfrak {X}} \rightarrow {\mathfrak {X}}$ of a chemical $x \in X$ is defined as the sum over all reactions catalyzed by *x*, i.e.

3.4 $$\begin{aligned} \phi _x = \sum _{(x,r) \in C} \phi _r. \end{aligned}$$

The multiplication $\circ$ is the usual composition of maps and therefore associative. The addition is associative, commutative and idempotent. The two operations $\circ$ and $+$ have obvious interpretations in terms of the function of enzymes on a CRS: The sum of two functions $\phi _x + \phi _y$ with $x,y \in X$ describes the *joint* or *simultaneous* function of two chemicals *x* and *y* on the CRS—in other words, the function $\phi _x + \phi _y$ acts on the set $X_F$ by yielding the products of all reactions which are catalyzed by *x*, *y*, or both (and analogously on any subset $Y \subset X_F$ for the reactions with all their reactants in $Y \cup F$). The composition of two functions $\phi _x \circ \phi _y$ with $x,y \in X$ describes their *subsequent* function: first *y* and then *x* act on the state space by their respective catalytic functions.

Recall that the *full transformation semigroup* ${\mathcal {T}}(A)$ of a finite discrete set *A* is the set of all maps $\{f:A \rightarrow A\}$, where the semigroup operation $\circ$ is the composition of maps.

#### Definition 3.2

Let (*X*, *R*, *C*, *F*) be a CRS. Its *semigroup model* ${\mathcal {S}}$ is the smallest subsemigroup of the full transformation semigroup ${\mathcal {T}}({\mathfrak {X}})$ closed under $\circ$ and $+$ that contains $\{\phi _x\}_{x \in X}$ and the zero function given by $0(Y) = \emptyset$ for all $Y \subset X_F$. The semigroup model is said to be *generated* by the set $\{\phi _x\}_{x \in X}$, indicated by the notation

$$\begin{aligned} {\mathcal {S}} = \langle \phi _x \rangle _{x \in X}. \end{aligned}$$

Note that this characterizes ${\mathcal {S}}$ *uniquely* as the intersection of all subsemigroups of ${\mathcal {T}}({\mathfrak {X}})$ which contain $\{\phi _x\}_{x \in X} \cup \{0\}$ and are closed under $\circ$ and $+$. Also, as a subsemigroup of ${\mathcal {T}}({\mathfrak {X}})$, the semigroup ${\mathcal {S}}$ is automatically a *finite* semigroup.

#### Remark 3.3

The object ${\mathcal {S}}$ is called a semigroup model, because ${\mathcal {S}}$ is a semigroup with respect to both operations $\circ$ and $+$. The correct description of ${\mathcal {S}}$ in terms of universal algebra is an algebra of type (2, 2, 0), cf. Almeida (1995). However, to avoid confusion with the more commonly used notions of an algebra (e.g. matrix algebras, operator algebras, algebras over rings, etc.), this terminology is not used. When referring to the actual semigroups $({\mathcal {S}},\circ )$ and $({\mathcal {S}},+)$, the term semigroup is used instead of semigroup model.

#### Remark 3.4

Rhodes and Nehaniv (2010) have introduced and studied similar semigroups derived from chemical reaction networks. These semigroups were constructed as subsemigroups of the full transformation semigroup on a set of metabolites induced by functions of enyzmes similar to the definition given here. However, in their work, the catalysts were not part of the network. More importantly, they did not define and use the operation of addition and also did not consider the natural partial order introduced in Remark 3.7. Therefore, most results of this article cannot be obtained with the construction of Rhodes and Nehaniv. Moreover, in the definition given here, the chemicals which are not produced by a function $\phi \in {\mathcal {S}}$ disappear after the application of the function to any $Y \in {\mathfrak {X}}$, whereas they are retained in the model of Rhodes and Nehaniv. Yet, this property is crucial for obtaining the results in Sect. 4.

#### Remark 3.5

Generally, a map $\phi : {\mathfrak {X}} \rightarrow {\mathfrak {X}}$ is to be defined on all subsets $Y \subset X_F$, i.e. the assignment $Y \mapsto \phi (Y)$ needs to be given independently for all $Y \subset X_F$. However, in the case of the constructed semigroup models, all maps $\phi \in {\mathcal {S}}$ respect the partial order on ${\mathfrak {X}}$ given by inclusion of sets, i.e.

3.5 $$\begin{aligned} Z \subset Y \subset X_F \implies \phi (Z) \subset \phi (Y) \end{aligned}$$

for all $Y,Z \subset X_F$. This follows directly from the Definition 3.1. Therefore, it is enough to specify any map $\phi \in {\mathcal {S}}$ on some set *I* of generating sets $\{Y_i\}_{i \in I}$ with $Y_i \subset X_F$ by explicitly giving $\phi (Y_i)$ for all $i \in I$ and by defining

$$\begin{aligned} \phi (Y) = \bigcup \limits _{Y_i \subset Y} \phi (Y_i) \end{aligned}$$

for an arbitrary $Y \subset X_F$. The $\{Y_i\}_{i \in I}$ and $\{\phi (Y_i)\}_{i \in I}$ are thereby required to satisfy the condition (3.5). Usually, the generators $\{Y_i\}_{i \in I}$ will be taken as the sets of non-food reactants involved in a given function $\phi \in {\mathcal {S}}$. This is a convenient notational simplification as the state space ${\mathfrak {X}}$ grows exponentially with the number of chemicals in the network. Finally, the following notation for constant maps is used throughout the text:

$$\begin{aligned} const_Y: {\mathfrak {X}} \rightarrow {\mathfrak {X}} \end{aligned}$$

denotes the map $const_Y(Z) = Y$ for all $Z \subset X_F$.

#### Example 3.6

**Fig. 3:**
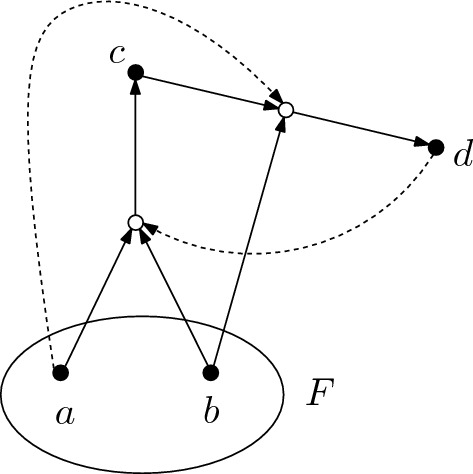
The CRS used to construct the semigroup model ${\mathcal {S}}$ in Example 3.6

As an example, the semigroup model ${\mathcal {S}}$ for the CRS shown in Fig. 3 is constructed.

This model is generated by the functions $\phi _a$ and $\phi _d$, whereby $\phi _a$ has the generating sets $\emptyset$ and $\{c\}$ with $\phi _a(\emptyset ) = \emptyset$ and $\phi _a(\{c\}) = \{d\}$ and $\phi _d$ is the constant function $const_{\{c\}}$. Therefore, the sum $\phi _a + \phi _d$ has two generating sets with $(\phi _a + \phi _d)(\emptyset ) = \{c\}$ and $(\phi _a + \phi _d)(\{c\}) = \{c,d\}$. Two additional functions are generated by the products $\phi _a \circ \phi _d = const_{\{d\}}$ and $(\phi _a + \phi _d)^2 = const_{\{c,d\}}$. One checks that these functions, together with 0 are closed under addition and multiplication. Thus the semigroup model ${\mathcal {S}}$ is the set

$$\begin{aligned} {\mathcal {S}} = \{0,\phi _a,\phi _d,\phi _a + \phi _d,const_{\{d\}},const_{\{c,d\}}\} \end{aligned}$$

equipped with the operations $\circ$ and $+$ given in Tables 1 and 2.

**Table 1:** The multiplication table for ${\mathcal {S}}$

$\circ$	$\phi _a$	$\phi _d$	$\phi _a + \phi _d$	$const_{\{d\}}$	$const_{\{c,d\}}$
$\phi _a$	0	$const_{\{d\}}$	$const_{\{d\}}$	0	$const_{\{d\}}$
$\phi _d$	$\phi _d$	$\phi _d$	$\phi _d$	$\phi _d$	$\phi _d$
$\phi _a + \phi _d$	$\phi _d$	$const_{\{c,d\}}$	$const_{\{c,d\}}$	$const_{\{c\}}$	$const_{\{c,d\}}$
$const_{\{d\}}$	$const_{\{d\}}$	$const_{\{d\}}$	$const_{\{d\}}$	$const_{\{d\}}$	$const_{\{d\}}$
$const_{\{c,d\}}$	$const_{\{c,d\}}$	$const_{\{c,d\}}$	$const_{\{c,d\}}$	$const_{\{c,d\}}$	$const_{\{c,d\}}$

The order of composition is *row* $\circ$ *column*

**Table 2:** The addition table for ${\mathcal {S}}$

+	$\phi _a$	$\phi _d$	$\phi _a + \phi _d$	$const_{\{d\}}$	$const_{\{c,d\}}$
$\phi _a$		$\phi _a + \phi _d$	$\phi _a + \phi _d$	$const_{\{d\}}$	$const_{\{c,d\}}$
$\phi _d$			$\phi _a + \phi _d$	$const_{\{c,d\}}$	$const_{\{c,d\}}$
$\phi _a + \phi _d$				$const_{\{c,d\}}$	$const_{\{c,d\}}$
$const_{\{d\}}$					$const_{\{c,d\}}$
$$const_{\{c,d\}}$$					

All functions $\phi$ satisfy $\phi + \phi = \phi$ giving the corresponding elements on the diagonal. The commutativity of addition yields the lower left half of the table

### Elementary properties

The interplay of the two operations on a semigroup model ${\mathcal {S}}$, the partial order on ${\mathcal {S}}$ and the partial order on the state space ${\mathfrak {X}}$ are all mutually compatible and the respective elementary properties are established in this section.

#### Remark 3.7

The partial order on ${\mathcal {S}}$ is given as follows: For $\phi , \psi \in {\mathcal {S}}$, the relation $\phi \le \psi$ holds if and only if ${\phi (Y) \subset \psi (Y)}$ for all $Y \subset X_F$. For ${\mathcal {S}}$ endowed with this partial order, the notation $({\mathcal {S}},\le )$ is used.

#### Lemma 3.8

Let ${\mathcal {S}}$ be a semigroup model of a CRS. The partial order defined above is preserved under the operations $\circ$ and $+$, i.e. for any $\phi , \phi ', \psi , \psi ' \in {\mathcal {S}}$ the following relations hold

3.6 $$\begin{aligned} \phi \le \psi \text { and } \phi ' \le \psi '&\Rightarrow \phi \circ \phi ' \le \psi \circ \psi ', \end{aligned}$$

3.7 $$\begin{aligned} \phi \le \psi \text { and } \phi ' \le \psi '&\Rightarrow \phi + \phi ' \le \psi + \psi '. \end{aligned}$$

#### Proof

This follows directly from the relation (3.5). $\square$

#### Lemma 3.9

Let ${\mathcal {S}}$ be a semigroup model of a CRS. Then the following properties hold true:

(I) Any $$\phi , \psi \in {\mathcal {S}}$$ satisfy

3.8 $$\begin{aligned} \phi \le \phi + \psi . \end{aligned}$$

(II) Any $\phi , \phi ', \psi \in {\mathcal {S}}$ such that $\phi \le \psi$ and $\phi ' \le \psi$ satisfy

3.9 $$\begin{aligned} \phi + \phi ' \le \psi . \end{aligned}$$

#### Proof

Both statements follow from the relation (3.7). To prove (I), one chooses $\phi ' = 0$. The statement (II) follows from the idempotence of addition with $\psi = \psi '$. $\square$

The operations $\circ$ and $+$ on ${\mathcal {S}}$ have the following distributivity properties.

#### Lemma 3.10

Let $\phi , \psi , \chi \in {\mathcal {S}}$. Then the following relations hold

3.10 $$\begin{aligned} \phi \circ \chi + \psi \circ \chi&= (\phi + \psi ) \circ \chi , \end{aligned}$$

3.11 $$\begin{aligned} \chi \circ \phi + \chi \circ \psi&\le \chi \circ (\phi + \psi ). \end{aligned}$$

#### Proof

Using the definitions of the operations, one obtains $(\phi \circ \chi + \psi \circ \chi )(Y) = (\phi \circ \chi )(Y) \cup (\psi \circ \chi )(Y) = \phi (\chi (Y)) \cup \psi (\chi (Y)) = (\phi + \psi )(\chi (Y)) = ((\phi + \psi ) \circ \chi )(Y)$ for all $Y \subset X$, proving the equality (3.10).

Lemma 3.8 and Lemma 3.9(I) imply $\chi \circ \phi \le \chi \circ (\phi + \psi )$ and $\chi \circ \psi \le \chi \circ (\phi + \psi )$. The relation (3.11) then follows from Lemma 3.9(II). $\square$

#### Remark 3.11

**Fig. 4:**
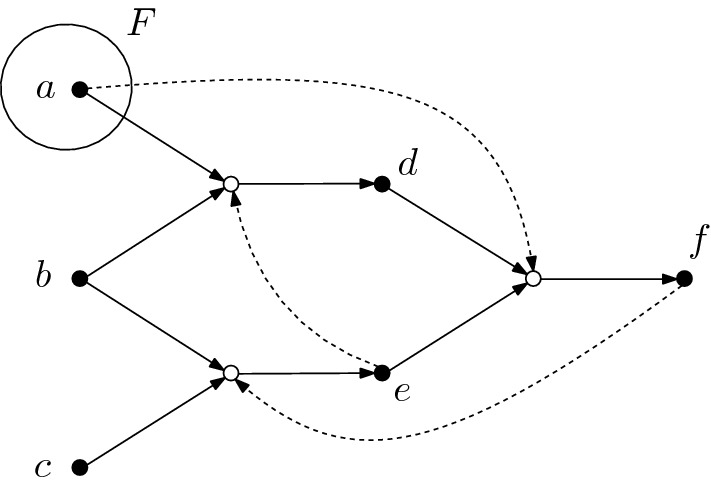
The semigroup model of the shown CRS has the property $\phi _a \circ (\phi _e + \phi _f) > \phi _a~\circ ~\phi _e~+~\phi _a~\circ ~\phi _f$

The inequality in (3.11) can be strict. In the example shown in Fig. 4, $\phi _a \circ \phi _e + \phi _a \circ \phi _f$ is the zero function, while the function $\phi _a \circ (\phi _e + \phi _f)$ is non-zero as can be seen by applying it to the set $\{b,c\}$, which gives $\phi _e(\{b,c\}) = \{d\}$ and $\phi _f(\{b,c\}) =\{e\}$ and therefore $(\phi _a \circ (\phi _e + \phi _f))(\{b,c\}) = \phi _a(\{d,e\}) = \{f\}$. The fact that the inequality (3.11) can be strict means that the result of applying a test function $\chi$ to the sum of two functions $\phi$ and $\psi$ can be larger than the result of applying the test function to the individual functions and then taking the sum. This is reminiscent of a characterization of emergence, which is often stated as *“the whole is larger than the sum of its parts”*.

By definition, the semigroup ${\mathcal {S}}$ captures all possibilities of joint and subsequent functions of chemicals on the state space ${\mathfrak {X}}$. In particular, this allows to determine the functions of arbitrary subsets $Y \subset X_F$ as follows.

#### Definition 3.12

For any $Y \subset X_F$, the semigroup model ${\mathcal {S}}(Y) < {\mathcal {S}}$ of the functions of *Y* is defined as^4^

$$\begin{aligned} {\mathcal {S}}(Y) = \langle \phi _x \rangle _{x \in Y \cup F} \end{aligned}$$

and the *function* $\Phi _Y$ of *Y* is given by

$$\begin{aligned} \Phi _{Y} = \sum _{\phi \in {\mathcal {S}}(Y)} \phi . \end{aligned}$$

The subsemigroup model ${\mathcal {S}}(Y)$ encodes all possible catalytic functionality that is supported on the set $Y \cup F \subset X$. The function $\Phi _Y$ is characterized by the following property.

#### Proposition 3.13

$\Phi _Y$ is the unique maximal element of ${\mathcal {S}}(Y)$.

#### Proof

It suffices to show that any element $\psi \in {\mathcal {S}}(Y)$ satisfies $\psi \le \Phi _{Y}$. But this is a direct consequence of Lemma 3.9 (I) as $\Phi _{Y} = \psi + \sum _{\phi \in {\mathcal {S}}(Y) {\setminus } \{\psi \}} \phi$ by construction. $\square$

#### Remark 3.14

If $Y \subset Z \subset X_F$, then Lemma 3.9(I) implies $\Phi _Y \le \Phi _Z$. In particular, ${\mathcal {S}}$ has a unique maximal element, given by $\Phi _{X_F}$.

#### Remark 3.15

Any subCRS $(X',R',C',F)$ has a semigroup model given by Definition 3.2. This model is denoted by ${\mathcal {S}}'(X',R')$. It is a subsemigroup of the full transformation semigroup ${\mathcal {T}}({\mathcal {P}}(X'_F))$ on the power set of $X'_F$. A function $\phi \in {\mathcal {S}}'(X',R')$ can be extended to a function $\text {ext}(\phi ) \in {\mathcal {T}}({\mathfrak {X}})$ by defining

$$\begin{aligned} \text {ext}(\phi )(Y) = \phi (Y \cap X_F') \end{aligned}$$

for all $Y \subset X_F$. This gives a homomorphic embedding of ${\mathcal {S}}'(X',R')$ into ${\mathcal {T}}({\mathfrak {X}})$, but $ext({\mathcal {S}}'(X',R'))$ is neither a subsemigroup of ${\mathcal {S}}(X'_F)$ nor of ${\mathcal {S}}$.

Because the generators $\{\phi '_x\}_{x \in X'} \subset {\mathcal {T}}({\mathcal {P}}(X'_F))$ of ${\mathcal {S}}'(X',R')$ and the generators $\{\phi _x\}_{x \in X'} \subset {\mathcal {T}}({\mathfrak {X}})$ of ${\mathcal {S}}(X'_F)$ satisfy $\text {ext}(\phi '_x) \le \phi _x$ for all $x \in X'$, the inequality

3.12 $$\begin{aligned} \text {ext}(\Phi '_{X'_F}) \le \Phi _{X'_F} \end{aligned}$$

for the maximal functions $\Phi '_{X'_F}$ and $\Phi _{X'_F}$ of ${\mathcal {S}}'(X',R')$ and ${\mathcal {S}}(X'_F)$ follows from Lemma 3.8.

## Characterization of self-sustaining CRS

In this section, it is illustrated how the semigroup model of a CRS provides a natural language to formulate and prove statements regarding CRS in a concise manner. The specific focus here lies on the application to self-sustaining CRS. In Sect. 4.1, it is shown that a CRS is self-sustaining if and only if its function $\Phi _{X_F}$ satisfies $\Phi _{X_F}(X_F) = X_F$ (Theorem 4.1) and it follows that any self-sustatining set of chemicals $X_F' \subset X_F$ satisfies $X'_F \subset \Phi _{X'_F}(X'_F)$ (Corollary 4.2). Moreover, the condition $X'_F = \Phi _{X'_F}(X'_F)$ is sufficient for $X'_F$ to be a self-sustaining set of chemicals (Proposition 4.3). This condition is, however, not necessary. In Sect. 4.2, a discrete dynamics is introduced by considering the repeated self-action of a set of chemicals, and the elementary properties of the dynamics are established. In Sect. 4.3 it is proven that the maximal self-sustaining set of chemicals of a CRS is the fixed point of the dynamics with the initial condition given by the presence of all chemicals of the CRS (Theorem 4.14). As a corollary, it follows that a CRS with a nilpotent semigroup model cannot have nontrival self-sustaining subCRS. This results establishes a link between the combinatorial theory of finite semigroups and the the theory of self-sustaining chemical reaction networks. Finally, a self-sustaining set of chemicals $X_F'$ is in general not stable under the dynamics but converges to a fixed point $X_F'^{*s}$ which contains $X_F'$. This fixed point is a self-sustaining set of chemicals itself and is termed the *functional closure* of $X_F'$. The importance of functionally closed sets of chemicals is discussed and a structure theorem on the set of all functionally closed sets of chemicals is proven (Theorem 4.26).

Throughout this section, fix a CRS (*X*, *R*, *C*, *F*) and let ${\mathcal {S}}$ be its semigroup model.

### Characterization of self-sustaining CRS

Recall that the self-sustainment property of a CRS in Definition 2.4 requires all chemicals in $X_F$ to be produced from chemicals in *X* by catalyzed reactions. Putting this description into mathematical language, one would expect a self-sustaining CRS to satisfy $\Phi _{X_F}(X_F) = X_F$. This is precisely the statement of the following theorem.

#### Theorem 4.1

A CRS is self-sustaining if and only if its maximal function $\Phi _{X_F}$ satisfies

$$\begin{aligned} \Phi _{X_F}(X_F) = X_F. \end{aligned}$$

#### Proof

If the CRS is self-sustaining, then there must be a set of reactions $R' \subset \pi _R(C)$ such that $\text {ran}(R') = X_F$. For each reaction $r \in R'$, choose a catalyst $y(r) \in X$. Then the function

$$\begin{aligned} \phi := \sum _{r \in R'} \phi _{y(r)} \end{aligned}$$

is an element of ${\mathcal {S}}$ and satisfies $\phi (X_F) = X_F$. The maximal function $\Phi _{X_F}$ is bounded from below by $\phi$ and therefore also satisfies $\Phi _{X_F}(X_F) = X_F$.

To prove the reverse, assume that $\Phi _{X_F}(X_F) = X_F$ holds. The function $\Phi _{X_F}$ must be of the form

$$\begin{aligned} \Phi _{X_F} = \sum _{y \in Y} \phi _y \circ \psi _y \end{aligned}$$

for some subset of generating functions $\{\phi _y\}_{y \in Y}$ with $Y \subset X$ and for some functions $\psi _y \in {\mathcal {S}}$,^5^. The function

$$\begin{aligned} \psi := \sum _{y \in Y} \phi _y \end{aligned}$$

satisfies $\psi (X_F) = X_F$, as otherwise $\Phi _{X_F}$ could not have $X_F$ in its image. Thus, the set of reactions

$$\begin{aligned} R' = \{ r \in R \text { such that }(y,r) \in C \text { for some }y \in Y\} \end{aligned}$$

satisfies the condition (S). $\square$

#### Corollary 4.2

If $X'_F \subset X_F$ is a self-sustaining set of chemicals, then the inclusion $X'_F \subset \Phi _{X'_F}(X'_F)$ holds.

#### Proof

Let $\Phi '_{X'_F}$ be the maximal function of the semigroup model ${\mathcal {S}}'(X',R\mid _{X'})$ for the subCRS generated by $X'_F$. By Theorem 4.1, the equality $\Phi '_{X'_F}(X'_F) = X'_F$ holds. Moreover, let $\Phi _{X'_F}$ be the maximal function of the subsemigroup model ${\mathcal {S}}(X'_F) < {\mathcal {S}}$. Then the inequality (3.12) yields the desired inclusion

$$\begin{aligned} X'_F = \text {ext}(\Phi '_{X'_F})(X'_F) \subset \Phi _{X'_F}(X'_F). \end{aligned}$$

$$\square $$

The inclusion $X'_F \subset \Phi _{X'_F}(X'_F)$ proven in the above corollary can be strict in the case that $X'$ contains catalysts that catalyze reactions with products outside of $X'$ but with reactants in $X'$. Therefore, the equality $X'_F = \Phi _{X'_F}(X'_F)$ cannot be a necessary condition for self-sustaining sets of chemicals. It is, however, a sufficient one:

#### Proposition 4.3

If a set of chemicals $X'_F \subset X_F$ satisfies the equality $X'_F = \Phi _{X'_F}(X'_F)$, then it is a self-sustaining set of chemicals.

#### Proof

The function $\Phi _{X'_F} \in {\mathcal {S}}(X'_F)$ is of the form

$$\begin{aligned} \Phi _{X'_F}&= \sum _{y \in Y} \phi _y \circ \psi _y \\&= \sum _{y \in Y} \sum _{(y,r) \in C} \phi _r \circ \psi _y \end{aligned}$$

for some subset of generating functions $\{\phi _y\}_{y \in Y}$ with $Y \subset X'$ and for some ${\psi _y \in {\mathcal {S}}(X'_F)}$. Define the function $\psi := \sum _{y \in Y} \psi _y$. The function $\Phi _{X'_F}$ is bounded above by $(\sum _{y \in Y} \phi _y) \circ \psi$, but due to its maximality in ${\mathcal {S}}(X'_F)$ it must be equal to this function, i.e. $\Phi _{X'_F} = (\sum _{y \in Y} \phi _y) \circ \psi$ with $\psi \in {\mathcal {S}}(X'_F)$, or, equivalently

4.1 $$\begin{aligned} \Phi _{X'_F} = \sum _{y \in Y} \sum _{(y,r) \in C} \phi _r \circ \psi . \end{aligned}$$

Moreover, the relation $\psi \le \Phi _{X'_F}$ holds, again due to the maximality of $\Phi _{X'_F}$, and this implies $\psi (X'_F) \subset X'_F$. This means that in the calculation of $\Phi _{X'_F}(X'_F)$ only those $\phi _r$ in the representation (4.1) with $\text {dom}(r) \subset \psi (X'_F) \cup F \subset X'$ play a role. In other words, choosing the set of reactions $R'$ as

$$\begin{aligned} R':= \left\{ r \in R \text { such that } (y,r) \in C \text { for some }y \in Y \text { and }\text {dom}(r) \subset X' \right\} , \end{aligned}$$

one can write $X'_F = \Phi _{X'_F}(X'_F)$ as

4.2 $$\begin{aligned} X'_F = \Phi _{X'_F}(X'_F) = \left( \sum _{r \in R'} \phi _r \circ \psi \right) \left( X'_F\right) = \bigcup _{r \in R'} \phi _r(\psi (X'_F)). \end{aligned}$$

This means that $X_F' = (\mathrm ran)(R')$ and thus the set $R'$ satisfies the condition (S). As $R' \subset R \mid _{X'}$, this implies that $X'_F$ is a self-sustaining set of chemicals. $\square$

#### Remark 4.4

Although the statement of Proposition 4.3 appears trivial at first glance, one needs to take care of the fact that $\Phi _{X'_F}$ is a function on the power set of $X_F$ and not on the power set of $X'_F$. Therefore, it could potentially include the formation of chemicals in $X'_F$ via reaction pathways which include intermediate chemicals outside of $X'$. The essence of the proof is to show, based on the maximality of $\Phi _{X'_F}$ in ${\mathcal {S}}(X'_F)$, that such pathways do not exist under the condition $X'_F = \Phi _{X'_F}(X'_F)$.

### Sustaining dynamics on a semigroup model

There is natural discrete dynamics on a CRS based on its semigroup model. Starting with any set of chemicals $Y_0 \subset X_F$, the maximal function $\Phi _{Y_0}$ (cf. Definition 3.12) acts on the set itself. This yields the maximal set $Y_1=\Phi _{Y_0}(Y_0)$ that can be produced from $Y_0$ and the food set using the functionality supported on $Y_0$ and the food set. This argument applies iteratively and gives rise to a discrete dynamical system on ${\mathfrak {X}}$.

#### Definition 4.5

The *sustaining dynamics*,^6^ of a CRS with the initial condition $Y_0 \subset X_F$ is generated by the propagator

$$\begin{aligned} {\mathcal {D}}^s : {\mathfrak {X}}&\rightarrow {\mathfrak {X}}\\ Y&\mapsto \Phi _Y(Y), \end{aligned}$$

where $\Phi _Y$ is the function of $Y \subset X_F$. Analogously, the dynamics is parametrized by ${\mathbb {Z}}_{\ge 0}$ as

$$\begin{aligned} Y_{n+1} = \Phi _{Y_n}(Y_n) \text { for all } n \in {\mathbb {Z}}_{\ge 0}. \end{aligned}$$

Note that the propagator ${\mathcal {D}}^s$ deletes all elements that are in *Y* but not in $\Phi _Y(Y)$.

#### Remark 4.6

Because the state space ${\mathfrak {X}}$ is finite, for the sequence $(Y_n)_{n \in {\mathbb {Z}}_{\ge 0}}$ generated by ${\mathcal {D}}^s$ under the initial condition $Y_0$, there exist minimal nonnegative integers *k* and $m \ne 0$ such that $Y_k = Y_{k+m}$. This gives rise to periodic behavior, i.e. $Y_{k+i} = Y_{k+i+nm}$ for all $i=0,...,m-1$ and all $n \in {\mathbb {N}}$. If $m=1$, then $Y_k$ is a fixed point and the dynamics is said to *stabilize* at $Y_k$. If $m>1$, the dynamics has period *m* and is *oscillatory*. Both behaviors are possible in CRS. The following notation for a fixed point will be often used.

#### Definition 4.7

A fixed point of the dynamics generated by ${\mathcal {D}}^s$ which results from the initial condition $Y_0$ is denoted by $Y_0^{*s}$.

#### Remark 4.8

According to Theorem 4.1, if the CRS is self-sustaining, then $X_F$ is a fixed point for the dynamics with initial condition $Y_0=X_F$.

#### Example 4.9

**Fig. 5:**
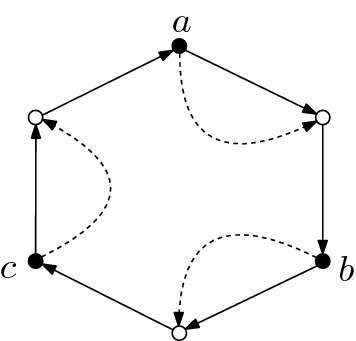
Example of a CRS with possible oscillatory dynamics. The food set is the empty set

Figure 5 shows a CRS with $X=\{a,b,c\}$, food set $F=\emptyset$ and the respective reactions shown in the figure. If the initial condition $Y_0$ is a proper subset of $X_F$, the dynamics has period 3. For example, the dynamics with the initial condition $Y_0=\{a\}$ is

$$\begin{aligned} \{a\} \mapsto \{b\} \mapsto \{c\} \mapsto \{a\} \mapsto ... \end{aligned}$$

The elementary properties of the sustaining dynamics are described in the following propositions.

#### Proposition 4.10

Let $(Y_n)_{n \in {\mathbb {Z}}_{\ge 0}}$ be the sustaining dynamics with initial condition $Y_0$. If the semigroup $({\mathcal {S}},\circ )$ of the CRS is nilpotent^7^, then the dynamics stabilizes at $\emptyset$, i.e. there exists a natural number *N* such that

$$\begin{aligned} Y_n = \emptyset \,\,\text { for all } n \ge N. \end{aligned}$$

#### Proof

By definition, $Y_n = \Phi _{Y_{n-1}} \circ \Phi _{Y_{n-2}} \circ ... \circ \Phi _{Y_0}(Y_0)$ holds. Because ${\mathcal {S}}$ is nilpotent, there exists an index *N* such that ${\mathcal {S}}^N = \{0\}$. This implies that $Y_n = \emptyset$ for all $n \ge N$. $\square$

A useful result is that the dynamics with initial condition $X_F$ cannot have periodic behavior, but always has a fixed point. It is a consequence of the following more general proposition.

#### Proposition 4.11

Let $(Y_n)_{n \in {\mathbb {Z}}_{\ge 0}}$ be the sustaining dynamics such that $Y_1 \subset Y_0$. Then

$$\begin{aligned} Y_{n+1} \subset Y_n \end{aligned}$$

holds for all $n \in {\mathbb {Z}}_{\ge 0}$ and the dynamics stabilizes at the fixed point $Y_0^{*s}$.

#### Proof

The proof proceeds by induction. By hypothesis $Y_1 \subset Y_0$ is satisfied. Let $Y_n \subset Y_{n-1}$. This implies the ordering of the respective functions $\Phi _{Y_n} \le \Phi _{Y_{n-1}}$ by Remark 3.14. Together with Remark 3.5 this yields

$$\begin{aligned} Y_{n+1} = \Phi _{Y_n}(Y_n) \subset \Phi _{Y_{n-1}}(Y_n) \subset \Phi _{Y_{n-1}}(Y_{n-1}) = Y_n. \end{aligned}$$

Thus the dynamics is given by a descending chain of sets

$$\begin{aligned} Y_0 \supset Y_1 \supset ... \supset Y_n \supset Y_{n+1} \dots \end{aligned}$$

Because $X_F$ is finite, the chain stabilizes. $\square$

#### Corollary 4.12

A dynamics $(Y_n)_{n \in {\mathbb {Z}}_{\ge 0}}$ with initial condition $Y_0 = X_F$ always leads to a fixed point.

#### Proof

This follows from $\Phi _{X_F}(X_F) \subset X_F$ and the previous proposition. $\square$

### Identification of self-sustaining sets of chemicals

In this section, the main theorem concerning the maximal self-sustaining set of chemicals of a CRS is stated and its biological significance is discussed. Its proof is merely a combination of the previously proven results.

The following lemma is required to ensure that the sustaining dynamics is well-behaved with respect to self-sustaining sets of chemicals.

#### Lemma 4.13

If $X'_F \subset X_F$ is a self-sustaining set of chemicals and if *Y* is a set that satisfies $X'_F \subset Y \subset X_F$, then the inclusion

$$\begin{aligned} X'_F \subset \Phi _Y(Y) \end{aligned}$$

holds.

#### Proof

The chain of inclusions

$$\begin{aligned} X'_F \subset \Phi _{X'_F}(X'_F) \subset \Phi _Y(X'_F) \subset \Phi _Y(Y) \end{aligned}$$

follows from the Corollary 4.2 and the Remarks 3.5 and 3.14. $\square$

Now the main theorem can be proven:

#### Theorem 4.14

(On the maximal self-sustaining set of chemicals). For any CRS, the maximal self-sustaining set of chemicals is the fixed point of the sustaining dynamics $(Y_n)_{n \in {\mathbb {Z}}_{\ge 0}}$ with the initial condition $Y_0 = X_F$, i.e. it is the set $X_F^{*s}$.

#### Proof

By Corollary 4.12, the dynamics stabilizes at a the fixed point $X_F^{*s}$. Being a fixed point, the set $X_F^{*s}$ satisfies

$$\begin{aligned} X_F^{*s} = \Phi _{X_F^{*s}}(X_F^{*s}) \end{aligned}$$

and is thus a self-sustaining set of chemicals by Proposition 4.3. Let $X'_F \subset X_F$ be any self-sustaining set of chemicals. By inductively applying the Lemma 4.13 to the sets $(Y_n)_{n \in {\mathbb {Z}}_{\ge 0}}$, it follows that $X'_F \subset X_F^{*s}$, which yields the maximality of $X_F^{*s}$. $\square$

#### Remark 4.15

The result of this theorem is closely related to an algorithm to determine the maximal pseudo-RAF set of reactions introduced by Hordijk et al. (2015). Therein, a dynamics $\{R_n\}_{n \in {\mathbb {Z}}_{\ge 0}}$ on the power set ${\mathcal {P}}(R)$ of reactions with the initial condition $R_0 = R$ is used. The propagator $R_n \mapsto R_{n+1}$ is defined by the operation

$$\begin{aligned} R_{n+1} = \{ r \in R_n \text { such that { r} has a catalyst in }\text {ran}(R_{n}) \cup F\}. \end{aligned}$$

This is equivalent to the dynamics $Y \mapsto \Phi _Y(Y)$ with the initial condition ${Y_0 = X_F}$ via the mutually inverse mapping $Y_n = \text {supp}(R_n)$ and ${R_n = \pi _{R\mid _{Y_n \cup F}}(C\mid _{Y_n \cup F})}$.

Theorem 4.14 has the following corollary.

#### Corollary 4.16

A CRS with a nilpotent semigroup $({\mathcal {S}},\circ )$ has no nontrivial self-sustaining subCRS.

#### Proof

If there was a nontrivial self-sustaining subCRS and thus a nonempty self-sustaining set of chemicals, there would be a maximal self-sustaining set of chemicals $X_F^{*s} \ne \emptyset$, which satisfies $X_F^{*s} = \Phi _{X_F^{*s}}(X_F^{*s})$. Any power $\Phi _{X_F^{*s}}^n$ of the function $\Phi _{X_F^{*s}}$ applied to $X_F^{*s}$ yields $X_F^{*s}$ and is therefore nonzero. $\square$

This corollary has importance when studying the semigroups that arise from self-sustaining CRS or CRS with nontrivial self-sustaining subCRS as it weeds out all nilpotent semigroups $({\mathcal {S}},\circ )$, which is the largest class of finite semigroups, cf. Satoh et al. (1994); Almeida (1995), as potential semigroup models.

### Functionally closed self-sustaining sets of chemicals

In Theorem 4.14, it was shown how to algorithmically identify the maximal self-sustaining set of chemicals as the fixed point $X_F^{*s}$ of the sustaining dynamics. For any given CRS, the self-sustaining subCRS are of importance to gain a deeper understanding of the modular structure the system. However, not all self-sustaining subCRS carry the same biological importance. For example, subCRS which are not closed according to Definition 2.5 should be considered as purely theoretical constructs because any subCRS occurring in the biophysical reality will always be closed. But the closure property as given in the Definition 2.5 only accounts for the closure on the level of reactions and not on the level of the functions of chemicals. This leads to the possibility that for a self-sustaining set of chemicals $X'_F \subset X_F$, the inclusion $X'_F \subset \Phi _{X'_F}(X'_F)$ is strict, i.e. that there are elements in $X_F {\setminus } X'_F$ which are produced from $X'$ by functionality supported on $X'$. Even more so, the inclusion $X'_F \subset \Phi _{X'_F}(X'_F)$ can hold for sets of chemicals which are not self-sustaining. A set $X'_F$ which is strictly included in $\Phi _{X'_F}(X'_F)$ will not be stable but will produce the chemicals $\Phi _{X'_F}(X'_F)$ over time. Then the chemicals in $\Phi _{X'_F}(X'_F)$ might catalyze the production of even more chemicals from $\Phi _{X'_F}(X'_F)$ and the food set. The following analogue of Proposition 4.11 ensures that this dynamics will lead to a fixed point.

#### Proposition 4.17

Let $(Y_n)_{n \in {\mathbb {Z}}_{\ge 0}}$ be the sustaining dynamics such that $Y_0 \subset Y_1$. Then $Y_{n} \subset Y_{n+1}$ holds for all $n \in {\mathbb {Z}}_{\ge 0}$ and the dynamics stabilizes at the fixed point $Y_0^{*s}$.

#### Proof

Analogous to the proof of Proposition 4.11. $\square$

Once the fixed point $X_F'^{*s}$ is reached, no further chemicals will be produced from the set $X_F'^{*s} \cup F$ by the functionality supported on it. By Proposition 4.3, the fixed point $X_F'^{*s}$ is a self-sustaining set of chemicals. Because $X_F'^{*s}$ contains the original set of chemicals $X'_F$, it should be thought of as the functional closure of $X'_F$. This suggests the following definitions.

#### Definition 4.18

A set of chemicals $X'_F \subset X_F$ which satisfies

$$\begin{aligned} X'_F \subset \Phi _{X'_F}(X'_F) \end{aligned}$$

is called *pre-functionally closed*.

A set of chemicals $X'_F \subset X_F$ is said to be *functionally closed* if it satisfies

$$\begin{aligned} X'_F = \Phi _{X'_F}(X'_F) \end{aligned}$$

The corresponding subCRS $(X',R\mid _{X'},C\mid _{X'},F)$ is said to be a *functionally closed subCRS*.

Let $\mathfrak {pF}$ denote the set of all pre-functionally closed sets of chemicals and ${\mathfrak {F}} \subset \mathfrak {pF}$ denote the set of all functionally closed sets of chemicals of the CRS:

$$\begin{aligned} \mathfrak {pF}&:= \{ X_F' \subset X_F \text { such that } X'_F \subset \Phi _{X'_F}(X'_F) \} \\ {\mathfrak {F}}&:= \{ X_F' \subset X_F \text { such that } X'_F = \Phi _{X'_F}(X'_F) \} \subset \mathfrak {pF}. \end{aligned}$$

#### Definition 4.19

For a pre-functionally closed set of chemicals $X'_F$, the fixed point of the sustaining dynamics $X_F'^{*s} \supset X'_F$ is called the *functional closure* of $X'_F$.

The notion of functional closure establishes another connection between the two sets $\mathfrak {pF}$ and ${\mathfrak {F}}$:

#### Lemma 4.20

The set ${\mathfrak {F}}$ is obtained by taking the functional closure of all elements of $\mathfrak {pF}$, i.e.

$$\begin{aligned} {\mathfrak {F}} = \{ Y^{*s} \text { for } Y \in \mathfrak {pF} \}. \end{aligned}$$

#### Proof

The set $\mathfrak {pF}$ contains all functionally closed sets and this does not change upon taking the functional closure, which shows the $\subset$ inclusion. The set on the right hand side contains only functionally closed sets of chemicals, thus the $\supset$ inclusion holds true. $\square$

These definitions have not been proposed in the CRS literature thus far to the best of my knowledge. One reason might be that they are cumbersome to formulate in the standard CRS terminology but become natural when the language of semigroup models is available. In the framework of chemical organization theory, however, there exists a notion of *closure* which is analogous to the notion of functional closure introduced here albeit it is expressed in a different mathematical language. See the review by Dittrich and Di Fenizio (2007) for more details on chemical organization theory.

The functionally closed sets of chemicals of any given CRS are important objects to understand the modular structure of the CRS, because they correspond to core functional modules of the CRS. Note that by Proposition 4.3, all functionally closed sets of chemicals are self-sustaining. Moreover, according to Corollary 4.2, all self-sustaining sets of chemicals are pre-functionally closed, which yields the following Theorem:

#### Theorem 4.21

For a set of chemicals to be self-sustaining, it is sufficient to be functionally closed and necessary to be pre-functionally closed, i.e., the following inclusions of sets hold true

$$\begin{aligned} \{\text {pre-functionally closed sets}\} \subset \{ \text {self-sustaining sets} \} \subset \{\text {functionally closed sets} \}. \end{aligned}$$

#### Proof

This follows directly from Corollary 4.2 and Proposition 4.3. $\square$

#### Example 4.22

**Fig. 8:**
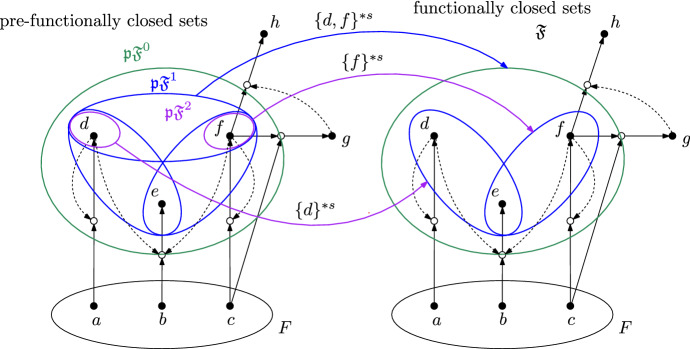
Illustration of the concept of (pre-)functional closure. The left hand side shows all pre-functionally closed sets *Y* of the CRN, i.e., sets which satisfy $Y \subset \Phi _Y(Y)$. In other words, these are the sets which are able to reproduce themselves (and possibly produce other additional chemicals) using only their own catalytic functionality. The set $\mathfrak {pF} = \cup _{i=0}^2 \mathfrak {pF}^i$ can be determined recursively as explained in the text. By applying the sustaining dynamics to each set in $\mathfrak {pF}$ until a fixed point is reached, one obtains the set ${\mathfrak {F}}$ of all functionally closed sets of chemicals, which is illustrated on the right hand side. These are the sets that reproduce themselves (and do not produce any additional chemicals) using only their own catalytic functionality. The sets $\{d\}, \{f\}$, and $\{d,f\}$ each produce the additional element *e* in the course of the dynamics and then stabilize. Thus they are pre-functionally closed but not functionally closed. The remaining sets $\{d,e,f\},\{d,e\}, \{e,f\}$ are functionally closed. The empty set is trivially functionally closed and therefore not displayed here (color figure online)

Consider the CRS shown in Fig. 1. The subCRS generated by $X'_F = \{c,d\}$ is self-sustaining, but not functionally closed as $\Phi _{X'_F}(X'_F) = X_F$ yields the set of all non-food chemicals, which is functionally closed. In fact, $X_F$ is the only nontrivial functionally closed set of chemicals of the given CRS. Another example in shown in Fig. 8 in “Appendix A”, with detailed explanations given in the figure caption.

It is possible to algorithmically determine the sets of all pre-functionally closed and functionally closed sets of chemicals of any given CRS. This is based on the following modification of the sustaining dynamics.

#### Definition 4.23

The *reduced sustaining dynamics* of a CRS with the initial condition $Y_0 \subset X_F$ is generated by the propagator

$$\begin{aligned} {\mathcal {D}}^{rs} : {\mathfrak {X}}&\rightarrow {\mathfrak {X}}\\ Y&\mapsto Y \cap \Phi _Y(Y). \end{aligned}$$

The trajectory $\{Y_n\}_{n \in {\mathbb {Z}}_{\ge 0}}$ of the dynamics is parametrized by ${\mathbb {Z}}_{\ge 0}$ as

$$\begin{aligned} Y_{n+1} = Y_n \cap \Phi _{Y_n}(Y_n) \text { for all } n \in {\mathbb {Z}}_{\ge 0}. \end{aligned}$$

#### Remark 4.24

Note that for the initial condition $Y_0 = X_F$, the reduced sustaining dynamics yields the same trajectory as the sustaining dynamics, because $\Phi _{Y_n}(Y_n) \subset Y_n$ is guaranteed by Proposition 4.11.

The reduced sustaining dynamics behaves well with respect to pre-functionally closed sets of chemicals:

#### Lemma 4.25

The reduced sustaining dynamics stabilizes for any initial condition $Y_0 \subset X_F$. Its fixed point, which is denoted by $Y_0^{*rs}$, is the maximal pre-functionally closed set of chemicals that is contained in $Y_0$.

#### Proof

By definition, the trajectory of the dynamics is given by a descending chain of sets $Y_0 \supset Y_1 \supset \dots \supset Y_n \supset Y_{n+1} \cdots$ and therefore it stabilizes. The fixed point satisfies the equation

$$\begin{aligned} Y_0^{*rs} = Y_0^{*rs} \cap \Phi _{Y_0^{*rs}}(Y_0^{*rs}), \end{aligned}$$

which is equivalent to $Y_0^{*rs} \subset \Phi _{Y_0^{*rs}}(Y_0^{*rs})$, i.e., $Y_0^{*rs}$ is pre-functionally closed.

Let $X'_F \subset Y_0$ be a pre-functionally closed set of chemicals contained in $Y_0$. By Lemma 4.13, it is contained in $\Phi _{Y_0}(Y_0)$ and thus in $Y_0 \cap \Phi _{Y_0}(Y_0)$. An inductive application of this argument proves that $X'_F \subset Y_n$ for all $n \in {\mathbb {Z}}_{\ge 0}$ and thus $X'_F \subset Y_0^{*rs}$, which shows the maximality of $Y_0^{*rs}$. $\square$

This suggests to define, for any $Y \subset X_F$, the set

$$\begin{aligned} {\mathfrak {p}}(Y):= \left\{ (Y{\setminus }\{y\})^{*rs} \right\} _{y \in Y} \subset {\mathfrak {X}} \end{aligned}$$

and, furthermore, to define iteratively

$$\begin{aligned} \mathfrak {pF}^0&:= \{ X_F^{*s} \} \\ \mathfrak {pF}^{i+1}&:= \bigcup _{Y \in \mathfrak {pF}^{i}} {\mathfrak {p}}(Y) \end{aligned}$$

for $i \in {\mathbb {Z}}_{\ge 0}$. This construction yields an (algorithmic) description of all pre-functionally closed sets of chemicals for any given CRN:

#### Theorem 4.26

(On the pre-functionally closed subsets of chemicals). There exists an $N \in {\mathbb {Z}}_{\ge 0}$ such that

$$\begin{aligned} \mathfrak {pF}^{i} = \{ \emptyset \} \end{aligned}$$

for all $i >N$. Moreover, the set

$$\begin{aligned} \mathfrak {pF}:= \bigcup _{i=0}^N \mathfrak {pF}^{i} \subset {\mathfrak {X}} \end{aligned}$$

is the set of all pre-functionally closed sets of chemicals.

#### Proof

Choose $N:= \mid X_F^{*s} \mid$. By construction, the cardinality $\mid Y \mid$ of a set $Y \in \mathfrak {pF}^{i}$ is at most $N -i$, which proves the first statement.

The maximal pre-functionally closed set of chemicals is $X_F^{*s}$ and it is contained in $\mathfrak {pF}$ by construction. Let $Y \subset X_F^{*s}$ be any pre-functionally closed set of chemicals. Then there exists a sequence of maximal length, which consists of pre-functionally closed sets

$$\begin{aligned} Y = Y_n \subsetneq Y_{n-1} \subsetneq \dots \subsetneq Y_1 \subsetneq X_F^{*s}. \end{aligned}$$

Then $Y_i \in \mathfrak {pF}^{i}$ by construction and thus $Y_n \in \mathfrak {pF}^{n}$ for some $n \le N$. Therefore, $\mathfrak {pF}$ contains all pre-functionally closed sets of chemicals. The reverse inclusion follows from Lemma 4.25, which states that all elements of $\mathfrak {pF}$ are pre-functionally closed sets of chemicals. $\square$

The set $\mathfrak {pF}$ can be constructed algorithmically and the Theorem 4.26 is thus applicable to the analysis of the CRS of real biological systems. Moreover, only the knowledge of the functions $\Phi _Y$ of sets of chemicals $Y \subset X_F$ is required to determine both $X_F^{*s}$ and $\mathfrak {pF}$. The knowledge of the full semigroup model is, on the contrary, not required. The set ${\mathfrak {F}}$ of all functionally closed sets is probably the most interesting object introduced in this work for practical applications. It is obtained, from $\mathfrak {pF}$, as the subset of fixed points of the sustaining dynamics, cf. Definition 4.19, or as the functional closure of $\mathfrak {pF}$ according to Lemma 4.20.

Functionally closed sets of chemicals should be important to gain insight into the modular structure of self-sustaining CRS. Moreover, this notion could be useful to analyze the evolutionary aspects of self-sustaining CRS. For example, if for a given chemical, there exists a unique minimal functionally closed set of chemicals containing it, then the chemical should be considered as a part of the module represented by the respective functionally closed set. If, however, there are multiple minimal functionally closed sets containing this chemical, then it should be considered as a mediator between these sets and would more likely have appeared when the respective modules were combined.

As discussed in the introductory paragraphs to this subsection, pre-functionally and functionally closed sets are more natural from the point of view of the discrete dynamics than the self-sustaining sets of chemicals. Theorem 4.21 shows, however, that the three notions are not independent but that self-sustaining sets are intermediate between the weaker notion of pre-functionally closed sets and the stronger notion of functionally closed sets.

This triad of sets is also interesting mathematically: There are lattice, bundle, and homological structures connecting them algebraically. This more abstract but rather elegant point of view is currently being prepared for publication.

## Discussion

In this work, it was shown that the CRS formalism has a natural algebraic structure induced by the simultaneous and subsequent functions of catalysts. The constructed semigroups contain all possible functional combinations and thus faithfully reflect the catalytic properties of the network. Moreover, the partial order on ${\mathcal {S}}$ allows to assign a well-defined catalytic function to any subset of chemicals. These functions respect the partial order on subsets of chemicals via inclusion and allow to define a sustaining and a reduced sustaining dynamics of the CRS. Finally, the interplay of these structures yields a characterization of the maximal self-sustaining set of chemicals. Moreover, a closer inspection of the sustaining dynamics naturally leads to the definition of (pre-)functionally closed sets of chemicals. Finally, a combination of the methods developed in this article allows to give a characterization of the sets of all (pre-)functionally closed sets of chemicals. Such sets of chemicals should play an important role in the analysis of the modular structure of CRS and pose valuable targets for the analysis of CRS corresponding to real biological systems, such as the ones constructed by Sousa et al. (2015) and Xavier and Kauffman (2022). Moreover, they bear interesting algebraic structures which lend themselves to future mathematical investigations.

Self-sustaining chemical reaction networks are an important area of research and this work introduces new techniques to field, which are based on the potentially powerful methods from semigroup theory. For example, Corollary 4.16 shows that a CRS with nilpotent semigroup cannot contain any nontrivial self-sustaining subCRS. This is an important fact by itself because most semigroups are nilpotent (any magma^8^ with the product of any three elements equal to zero is automatically a semigroup) and this weeds out these objects in the study of self-sustaining networks. In semigroup theory, combinatorial problems are an important and developed field and with the construction provided in this article, such methods can now be applied to the combinatorics of self-sustaining CRS. However, all central results given here require a representation of the semigroup model as a subsemigroup of the full transformation semigroup ${\mathcal {T}}({\mathfrak {X}})$ and are based on particular properties of this representation. It would be interesting to find purely algebraic descriptions of semigroup models of CRS and to restate the main results purely algebraically, i.e. without reference to this representation.

Moreover, there is an equivalence between finite semigroups and finite automata established by Schützenberger (1965). Thus the semigroup models developed here suggest to investigate the computational capabilities of catalytic reaction systems as a future direction of research. In this regard, it is interesting to study the inverse problem, i.e. to determine which finite semigroups can be realized as semigroup models of CRS and to analyze their computational properties. It is clear that not all semigroups can be interpreted as semigroup models of some CRS, because for a general finite semigroup, a partial order satisfying Lemma 3.8 does not exist.

Further interesting questions in this direction arise for the semigroups of infinite reaction networks Steel (2015) and the classification of their computational properties. The definitions given here extend directly to infinite networks, but the arguments based on finiteness of ${\mathcal {S}}$ and ${\mathfrak {X}}$ used in many proofs then require modification and in some cases the analogous results do not hold. For example, the discrete dynamics can lead to a steadily growing network instead of a fixed point or periodic orbit. Another possibility is the extension of the state space from $\{0,1\}^{X_F}$ to ${\mathbb {R}}_{\ge 0}^{X_F}$ by taking into account the concentrations of the chemicals. In this case, the dynamics is governed by the classical kinetic rate equations and if one could establish a natural link from the classical models to a CRS description, one would be led to the notion of function and causality for such classical models.

The main motivation for the construction of algebraic models for chemical reaction networks is a natural possibility of coarse-graining through taking quotients by congruences. In this approach, the possible coarse-graining procedures are given by the lattice of congruences on the algebraic structure. This has the advantage that the coarse-grained model is naturally equipped with the same structure as the original model and that consecutive coarse-graining procedures over several scales are feasible. This approach will be investigated in detail in a forthcoming publication.

Finally note that self-generating CRS, which are called RAF in the literature, can be characterized in an analogous fashion as presented for self-sustaining CRS in this work. The only difference is that the sustaining dynamics $Y \mapsto \Phi _Y(Y)$ needs to be replaced by the generative dynamics $Y \mapsto \Phi _Y(\emptyset )$. However, the proofs require a deeper understanding of the structure of the semigroups, which is provided in a companion article by Loutchko (2023).

## Figures and Tables

**Fig. 6:**
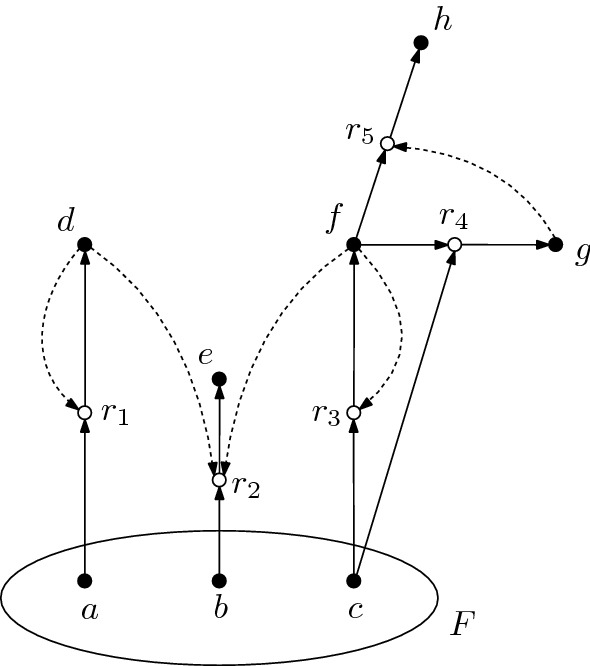
Example of a CRS. The CRS consists of eight chemicals $X=\{a,b,c,d,e,f,g,h\}$ and five reactions $r_1:a\rightarrow d$, $r_2:b \rightarrow e$, $r_3: c \rightarrow f$, $r_4:c + f \rightarrow g$, and $r_5: f \rightarrow h$. The reaction $r_1$ is catalyzed by *c*, the reaction $r_2$ is catalyzed by *d* or *f*, the reaction $r_3$ is catalyzed by *f*, the reaction $r_5$ is catalyzed by *g*, and the reaction $r_4$ is not catalyzed. The food set is given by $F = \{a,b,c\}$

**Fig. 7:**
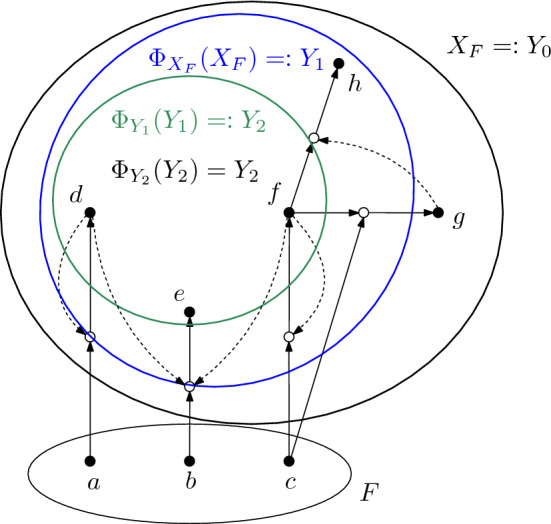
Sustaining dynamics $Y \mapsto \Phi _Y(Y)$ for the identification of the largest self-sustaining set of chemicals of the CRS. Starting with the full set $Y_0:= X_F$ (black circle), the set acts on itself by its function $\Phi _{X_F} = \phi _d + \phi _f + \phi _g \circ \phi _f$ which yields $Y_1:= \Phi _{X_F}(X_F) = \{d,e,f,h\}$ (blue circle). The chemical *g* is missing from $Y_1$ because it is not produced by any catalyzed reaction. In the next step, $Y_1$ acts on itself which gives $Y_2:= \Phi _{Y_1}(Y_1) = \{d,e,f\}$. Here, the chemical *h* is not produced anymore because the respective catalyst, *g*, is not in $Y_2$. Finally, the self-action of $Y_2$ reproduces $Y_2 = \Phi _{Y_2}(Y_2)$. The fixed point equation $Y_2 = \Phi _{Y_2}(Y_2)$ implies that the set is self-sustaining. Because the initial condition was chosen to be the whole set $X_F$, this fixed point also must be the largest self-sustaining set (color figure online)

## Appendix A: Example complementing the mathematical outline

Each of the following paragraphs illustrates the concepts from the corresponding paragraph in the Mathematical Outline. Following through this example should give the reader a good intuition of what is happening in this article, without getting lost in technical details.

**The CRS formalism** Figure 6 shows an example of a CRS. The solid disks represent the chemicals *X*, circles represent reactions *R*, solid directed edges represent reactants and products of a reaction, and dashed edges from *x* to *r* represent catalysis data $(x,r) \in C$. The food set *F* is represented by a circle around the respective chemicals.

**The semigroup model of a CRS** The construction of the semigroup model for the CRS from Fig. 6 is sketched here. The set of non-food chemicals is $X_F = \{d,e,f,g,h\}$ and the state space is its power set ${\mathfrak {X}} = {\mathcal {P}}(X_F)$, which consists of all subsets of $X_F$ and therefore has $2^5 = 32$ elements. By definition, the functions $\phi _{r_1},\phi _{r_2},\phi _{r_3}: {\mathfrak {X}} \rightarrow {\mathfrak {X}}$ are the constant functions given by

$$\begin{aligned}&\phi _{r_1}(Y) = \{d\} \\&\phi _{r_2}(Y) = \{e\} \\&\phi _{r_3}(Y) = \{f\} \end{aligned}$$

for any $Y \subset X_F$. The function $\phi _{r_4}(Y)$ gives $\{g\}$ if and only if $f \in Y$, and returns the empty set $\emptyset$ otherwise. Similarly, $\phi _{r_5}(Y)$ gives $\{h\}$ if and only if $f \in Y$ and $\emptyset$ otherwise. The functions of the chemicals are then given by the sum of reactions which they catalyze, which yields two constant functions

$$\begin{aligned}&\phi _d = (\phi _{r_1} + \phi _{r_2})(Y) = \{d,e\} \\&\phi _f = (\phi _{r_2} + \phi _{r_3})(Y) = \{e,f\} \end{aligned}$$

for any $Y \subset X_F$ and the function $\phi _g = \phi _{r_5}$. Note that the chemicals *e* and *h* have no functions attached to them and that the function $\phi _{r_4}$ does not appear in any of the functions of the chemicals because it is not catalyzed. The semigroup model ${\mathcal {S}}$ is generated by the three functions $\phi _d,\phi _f$, and $\phi _g$. Without listing the complete Cayley tables for addition and multiplication (this is done for another toy CRS in Example 3.6 in the main text), the operations are illustrated: The summation of functions means that all reactions take place that are contained within one or the other function, for example the sum $(\phi _d + \phi _g)(Y)$ will give $\{d,e,h\}$ if $f \in Y$ and $\{d,e\}$ otherwise. The product should be thought of as carrying out functions subsequently, i.e., the function on the right should produce the reactants of the function to its left in order to give non-trivial composition. For example, the product $(\phi _g \circ \phi _f)(Y)$ will yield $\{h\}$ for all $Y \subset X_F$ because $\phi _f$ will always produce *f*, which acts as the reactant for $\phi _g$. In contrast, $(\phi _g \circ \phi _d)(Y)$ will always yield $\emptyset$ because the reactant for $\phi _g$ is not produced by its preceding function $\phi _d$.

The partial order of functions requires the larger function to always produce at least the same chemicals from the same set *Y* as the smaller function. For example, $\phi _d \le \phi _d + \phi _g$ holds because the left hand side is the constant function which always yields $\{d,e\}$ and $\phi _d + \phi _g$ either gives $\{d,e\}$ or $\{d,e,h\}$. One can verify that the function $\phi _d + \phi _f + \phi _g \circ \phi _f$ is the maximal function of ${\mathcal {S}}$, it is given by $(\phi _d + \phi _f + \phi _g \circ \phi _f)(Y) = \{d,e,f,h\}$ for all $Y \subset X_F$. Taking the subset $\{d,g\} \subset X_F$, the semigroup model ${\mathcal {S}}(\{d,g\})$ supported on this subset is generated by the two functions $\phi _d$ and $\phi _g$. One verifies that ${\mathcal {S}}(\{d,g\})$ consists of the four functions $\{0,\phi _d,\phi _g,\phi _d + \phi _g\}$, where $0(Y) = \emptyset$ for all $Y \subset X_F$, and that it has $\Phi _{\{d,g\}} = \phi _d + \phi _g$ as its maximal element.

**Characterization of self-sustaining CRS** In the example from Fig. 6, the maximal function $\Phi _{X_F}$ is given by $\Phi _{X_F} = \phi _d + \phi _f + \phi _g \circ \phi _f$ and does not satisfy $\Phi _{X_F} (X_F)= X_F$, i.e., $X_F$ is not a self-sustaining set of chemicals. The reason is that *g* cannot be formed by a catalyzed reaction and thus the catalyst for the reaction $r_5$ cannot be part of a self-sustaining set of chemicals, which in turn means that *h* cannot be formed eventually. This is reflected in the dynamics $Y_0:= X_F = \{d,e,f,g,h\} \mapsto \Phi _{X_F}(X_F) = \{d,e,f,h\} =: Y_1 \mapsto \Phi _{Y_1}(Y_1) = \{d,e,f\} =: Y_2$, where first *g* and then *h* disappears (the functions $\Phi _{Y_1}$ and $\Phi _{Y_2}$ of the sets $Y_1 = \{d,e,f,h\}$ and $Y_2 \{d,e,f\}$ are actually equal, as *g* has no catalytic functionality, and are both given by $\Phi _{Y_1} = \Phi _{Y_2} = \phi _d + \phi _f$). This dynamics is depicted in Fig. 7. The remaining set $Y_2 = \{d,e,f\}$ is self-sustaining, because all chemicals in this set can be formed from the food set by using catalytic functionality which is supported on this set. Mathematically, this is expressed through the fixed point equation $\Phi _{Y_2}(Y_2) = Y_2$.

Also note that the CRS contains other smaller self-sustaining sets of chemicals, namely $\{d,e\},\{e,f\},\{d,f\},\{d\},\{f\}$, and the empty set $\emptyset$ (the latter being the trivial case of self-sustainment).

**Functionally closed self-sustaining sets of chemicals** The recursive construction of the set $\mathfrak {pF}$ of pre-functionally closed sets starts with the maximal self-sustaining set of chemicals $\mathfrak {pF}^0 = \{ X_F^{*s} \} = \{\{d,e,f\}\}$, and by individually removing chemicals from $X_F^{*s}$ and applying the reduced dynamics to the resulting two element sets one finds that all of them are pre-functionally closed, i.e., $\mathfrak {pF}^1 = \{\{d,e\},\{d,f\},\{e,f\}\}$. For the second iteration, elements from the sets in $\mathfrak {pF}^1$ are individually removed and the resulting one-element sets undergo the reduced dynamics: The set $\{e\}$ decays to $\emptyset = \{e\}^{*rs}$ because *e* has no catalytic functionality and the other two sets $\{d\}$ and $\{f\}$ survive. This gives $\mathfrak {pF}^2 = \{\{d\},\{f\},\emptyset \}$. For $i \ge 3$, one has $\mathfrak {pF}^i = \{\emptyset \}$. The pre-functionally closed sets of chemicals are thus $\{d,e,f\},\{d,e\},\{d,f\},\{e,f\},\{d\},\{f\}$, and $\emptyset$. This is shown on the left in Fig. 8.

Then, applying the characterization of ${\mathfrak {F}}$ as the functional closure of all sets in $\mathfrak {pF}$, one checks that *d* and *f* each produce *e* and then stabilize, thus $\{d\}^{*s} = \{d,e\}$, $\{f\}^{*s} = \{e,f\}$, and $\{d,f\}^{*s} = \{d,e,f\}$ which shows that the three sets $\{d\}, \{f\}$, and $\{d,f\}$ are pre-functionally closed but not functionally closed. The functionally closed sets are $\{d,e,f\},\{d,e\}, \{e,f\}$, and $\emptyset$. This is also illustrated in Fig. 8.

## Appendix B: List of mathematical symbols

See Table 3.

**Table 3:** List of mathematical symbols used in the article

Symbol	Description	Definition/first appearance
(*X*, *R*, *C*, *F*)	A CRS	Definition 2.1
$\text {dom}(r),\text {dom}(R')$	Domain (set of substrates) of a reaction *r* (of a set $R'$ of reactions)	Section 2.1
$\text {ran}(r),\text {ran}(R')$	Range (set of products) of a reaction *r* (of a set $R'$ of reactions)	Section 2.1
$\text {supp}(r),\text {supp}(R')$	Support (set of substrates and products) of a reaction *r* (of a set $R'$ of reactions)	Section 2.1
$X_F$	Set of non-food chemicals	Section 2.1
$\pi _R:C \rightarrow R$	Projection $(x,r) \mapsto r$	Equation 2.1
$(X',R',C',F)$	A subCRS of (*X*, *R*, *C*, *F*)	Definition 2.3
$R\mid _{X'}$	Set of reactions supported on $X'$	Definition 2.3
$C\mid _{X'}$	Catalysis data supported on $X'$	Definition 2.3
$CRS(X'_F)$	subCRS generated by $X'_F$	Equation 2.2
${\mathfrak {X}}$	State space of the CRS	Section 3.1
${\mathcal {P}}(A)$	Power set of a set *A*	Section 3.1
$\phi _r,\phi _x$	Function of the reaction *r*, of a chemical *x*	Definition 3.1
${\mathcal {T}}(A)$	Full transformation semigroup of a set *A*	Section 3.1
${\mathcal {S}}$	Semigroup model of the CRS	Definition 3.2
0	Zero function $0 \in {\mathcal {S}}$	Definition 3.2
$const_Y: {\mathfrak {X}} \rightarrow {\mathfrak {X}}$	Constant function $const_Y(Z) = Y$	Section 3.1
$({\mathcal {S}},\le )$	Semigroup model equipped with the partial order	Remark 3.7
${\mathcal {S}}(Y)$	Semigroup model generated by the functions of $Y \subset X$	Definition 3.12
$\Phi _{Y}$	The function of the set *Y*	Definition 3.12
${\mathcal {S}}'(X',R\mid _{X'})$	Semigroup model of the subCRS ${(X',R\mid _{X'},C\mid _{X'},F)}$	Remark 3.15
*ext*	Extension of functions ${\mathcal {T}}({\mathcal {P}}(X'_F)) \rightarrow {\mathcal {T}}({\mathfrak {X}})$	Remark 3.15
${\mathcal {D}}^s$	Generator of the sustaining dynamics	Definition 4.5
$Y_0^{*s}$	Fixed point of the sustaining dynamics	Definition 4.7
$\mathfrak {pF}$	Pre-functionally closed sets of chemicals	Definition 4.18
${\mathfrak {F}}$	Functionally closed sets of chemicals	Definition 4.18
${\mathcal {D}}^{rs}$	Reduced sustaining dynamics	Definition 4.23
$Y_0^{*rs}$	Fixed point of the reduced sustaining dynamics	Lemma 4.25
${\mathfrak {p}}: {\mathfrak {X}} \rightarrow {\mathcal {P}}({\mathfrak {X}})$	Map giving maximal pre-functionally closed sets of chemicals	Section 4.4

The first appearance refers to the main text and not to the mathematical outline

## Footnotes

1 Usually, a net chemical reaction is denoted as $r: a_1 A_1 + a_2 A_2 +.. + a_n A_n \longrightarrow b_1 B_1 + b_2 B_2 +... + b_m B_m$, where $a_i,b_j \in {\mathbb {N}}$ and $A_i,B_j \in X$ such that $A_i \ne B_j$ for all $i=1,...,n$ and $j=1,...,m$. In the definition used here, the stoichiometric information is discarded and $\text {dom}(r) = \{A_i\}_{i=1}^n$ and $\text {ran}(r) = \{B_j\}_{j=1}^m$ are used instead.

2 This can be made precise by saying that two subCRS $(X',R'_1,C'_1,F)$ and $(X',R'_2,C'_2,F)$ with the same set of chemicals $X'$ are catalytically equivalent if $\pi _{R'_1}(C'_1) = \pi _{R'_2}(C'_2)$ holds. The catalytical equivalence classes of subCRS are ordered by applying the partial order of subCRS to the minimal representatives of each catalytical equivalence class.

3 Note that although *Y* does not contain *F*, the constant presence of the food set is accounted for in the definition via formula (3.1).

4 Due to the constant presence of the food set, the functions of the chemicals in *F* are included in the set of generators for ${\mathcal {S}}(Y)$.

5 Note that, *a priori* *Y* should be a *multiset* with elements in *X* and the argument of the proof would go through unaltered if *Y* was a multiset. However, using the relation (3.11), i.e. $\phi _y \circ \psi _y + \phi _y \circ \psi '_y \le \phi _y \circ (\psi _y + \psi '_y)$, and the maximality of $\Phi _{X_F}$, one sees that the given representation of $\Phi _{X_F}$ with a *set* *Y* is actually correct.

6 Instead of *dynamics* the more commonly used term would be *discrete dynamical system*. However, using the more common terminology would make the text more cumbersome to read and therefore *dynamics* is used from now on.

7 Recall that a semigroup $\mathcal {S}$ is *nilpotent* if the ideal $\mathcal {S}^n := \{ \phi _1 \circ \phi _2 \circ \cdots \circ \phi _n \vert \phi _i \in \mathcal {S} \}$ becomes trivial, i.e., if $\mathcal {S}^n = \{ 0 \}$ for some $n \in \mathbb {N}$.

8 A magma is a semigroup without the associativity property.

## References

[CR1] Almeida J (1995). Finite semigroups and universal algebra.

[CR2] Bonchev DD, Mekenyan OG (2012). Graph theoretical approaches to chemical reactivity.

[CR3] Dittrich P, Di Fenizio PS (2007). Chemical organisation theory. Bull Math Biol.

[CR4] Dyson F (1999). Origins of life.

[CR5] Eigen M (1971). Selforganization of matter and the evolution of biological macromolecules. Naturwissenschaften.

[CR6] Gánti T (1975). Organization of chemical reactions into dividing and metabolizing units: the chemotons. Biosystems.

[CR7] Gilbert W (1986). Origin of life: the RNA world. Nature.

[CR8] Hordijk W, Steel M (2004). Detecting autocatalytic, self-sustaining sets in chemical reaction systems. J Theor Biol.

[CR9] Hordijk W, Steel M (2017). Chasing the tail: the emergence of autocatalytic networks. Biosystems.

[CR10] Hordijk W, Steel M (2018). Autocatalytic networks at the basis of life’s origin and organization. Life.

[CR11] Hordijk W, Kauffman SA, Steel M (2011). Required levels of catalysis for emergence of autocatalytic sets in models of chemical reaction systems. Int J Mol Sci.

[CR12] Hordijk W, Hasenclever L, Gao J (2014). An investigation into irreducible autocatalytic sets and power law distributed catalysis. Nat Comput.

[CR13] Hordijk W, Smith JI, Steel M (2015). Algorithms for detecting and analysing autocatalytic sets. Algorithms Mol Biol.

[CR14] Hordijk W, Steel M, Kauffman SA (2019). Molecular diversity required for the formation of autocatalytic sets. Life.

[CR15] Joyce GF (1989). RNA evolution and the origins of life. Nature.

[CR16] Kauffman SA (1971). Cellular homeostasis, epigenesis and replication in randomly aggregated macromolecular systems. J Cybern.

[CR17] Kauffman SA (1986). Autocatalytic sets of proteins. J Theor Biol.

[CR18] Kauffman SA (1993). The origins of order: self-organization and selection in evolution.

[CR19] Loutchko D (2023) An algebraic characterization of autocalatyic sets using semigroup models. J Math Biol. 10.1007/s00285-023-01899-4 PMC10113333 37071214

[CR20] Oparin AI (1957). The origin of life on the earth.

[CR21] Rhodes J, Nehaniv CL (2010). Applications of automata theory and algebra: via the mathematical theory of complexity to biology, physics, psychology, philosophy, and games.

[CR22] Rosen R (1958). A relational theory of biological systems. Bull Math Biol.

[CR23] Satoh S, Yama K, Tokizawa M (1994) Semigroups of order 8. In: Semigroup forum. Springer, pp 7–29

[CR24] Schützenberger MP (1965). On finite monoids having only trivial subgroups. Inf Control.

[CR25] Sousa FL, Hordijk W, Steel M (2015). Autocatalytic sets in *E. coli* metabolism. J Syst Chem.

[CR26] Steel M (2015). Self-sustaining autocatalytic networks within open-ended reaction systems. J Math Chem.

[CR27] Steel M, Hordijk W, Smith J (2013). Minimal autocatalytic networks. J Theor Biol.

[CR28] Varela FG, Maturana HR, Uribe R (1974). Autopoiesis: the organization of living systems, its characterization and a model. Biosystems.

[CR29] Vasas V, Fernando C, Santos M (2012). Evolution before genes. Biol Direct.

[CR30] von Neumann J (1966). Theory of self-reproducing automata. Math Comput.

[CR31] Xavier JC, Kauffman S (2022). Small-molecule autocatalytic networks are universal metabolic fossils. Philos Trans R Soc A.

[CR32] Xavier JC, Hordijk W, Kauffman S et al (2020) Autocatalytic chemical networks at the origin of metabolism. Proc R Soc B 287(1922):20192,377 10.1098/rspb.2019.2377 PMC7126077 32156207

